# “Getting to diagnosis was an absolute nightmare”: survey insights about the lived experience of spinal CSF leak in Australia and Aotearoa New Zealand

**DOI:** 10.1007/s00415-026-13840-y

**Published:** 2026-04-30

**Authors:** Lachlan S. W. Knight, Rachel L. Smith, Alexis Ceecee Britten-Jones, Sam E. John, David B. Grayden, Bang V. Bui, Lauren N. Ayton, Bao N. Nguyen

**Affiliations:** 1 Department of Optometry and Vision Sciences, The University of Melbourne, Parkville, Victoria Australia; 2 College of Medicine and Public Health, Flinders Health and Medical Research Institute, Flinders University, Adelaide, South Australia Australia; 3 Spinal CSF Leak Australia and CSF Leakers DownUnder Patient Support Group, Milton, Queensland Australia; 4 Centre for Eye Research Australia, Royal Victorian Eye and Ear Hospital, Melbourne, Victoria Australia; 5 Department of Biomedical Engineering, Graeme Clark Institute for Biomedical Engineering, The University of Melbourne, Parkville, Victoria Australia; 6 Department of Medicine, The University of Melbourne, Parkville, Victoria Australia; 7 Department of Surgery (Ophthalmology), The University of Melbourne, Parkville, Victoria Australia

**Keywords:** Cerebrospinal fluid leak, Spinal CSF leak, Intracranial hypotension, Orthostatic headache, Quality of life

## Abstract

**Background:**

Spinal cerebrospinal fluid (CSF) leak is a disabling and often misdiagnosed condition characterised by CSF hypovolemia. Associated neurological symptoms are diverse and often leave individuals bed-bound due to their orthostatic nature. Prior literature describing the difficulties in diagnosis, treatment, and ongoing impact of CSF leak is, thus far, confined to Europe and North America. This study provides a novel account of lived experiences of spinal CSF leak in Australia and Aotearoa New Zealand (NZ).

**Methods:**

An online survey exploring symptoms, diagnosis, treatment, and effect on daily life of a person’s “first” CSF leak was designed with consumer involvement. Responses were received from May to August 2025. Open-text responses were analysed using thematic analysis.

**Results:**

In total, 106 surveys were completed. Over 70 symptoms were reported; the most common were orthostatic headache (95.3%), neck pain (85.8%), and brain fog (79.2%). Most people considered their diagnosis (73.6%) and treatment (65.3%) difficult, underscored by limited clinician awareness and access to care, leaving individuals to self-advocate. Amongst symptomatic participants (73.6%), median EuroQol Visual Analogue Scale score was 40 (interquartile range 25–64; indicating low health-related quality-of-life) and mean Headache Impact Test-6 score was 69 ± 5 (indicating severe impact). Other challenges identified included navigating change to social identity and daily functioning.

**Conclusions:**

The spinal CSF leak experience in Australia and NZ is comparable to reports from other high-income countries, highlighting the global need to increase awareness of spinal CSF leak, support timely diagnostic, referral and treatment pathways, and mitigate its impact on quality of life.

**Supplementary Information:**

The online version contains supplementary material available at 10.1007/s00415-026-13840-y.

## Introduction

Spinal cerebrospinal fluid (CSF) leak is a debilitating condition characterised by low intracranial volume due to extradural CSF collections or CSF-venous fistulas in the spinal column [1, 2]. A spinal CSF leak may occur spontaneously or secondary to trauma or a spinal procedure (e.g., epidural anaesthesia) [3, 4]. The condition is associated with a myriad of neurological symptoms, with almost all individuals experiencing orthostatic headache [1]. Nausea and vomiting, dizziness, neck pain, and tinnitus are other relatively common symptoms, typically relieved with prolonged periods of recumbency [1, 5]. Individuals are, therefore, often bed-bound, leading to immense disability and profound impact on their quality of life [6].

Diagnostic delays and misdiagnosis occur frequently, as spinal CSF leak can be profoundly challenging to diagnose [7]. Spinal CSF leak has an overall estimated annual incidence of 3.8–4.6 per 100,000 in the adult population, based on data from two predominantly Caucasian populations from tertiary healthcare centres in North America [8, 9]. This figure is likely an underestimation due to patient setting, diagnostic criteria used, and imaging techniques available. Current diagnostic criteria [10] require neuroimaging evidence of extradural CSF (detected in only 48–76% of individuals) or a low CSF pressure obtained through lumbar puncture (an invasive and often unsuccessful procedure that can aggravate symptoms) [1]. Recent global consensus guidelines recommend against performing a lumbar puncture, with preference to perform magnetic resonance imaging with intravenous contrast of the brain and spine instead [11]. More advanced imaging techniques, including digital subtraction myelography and magnetic resonance myelography with intrathecal gadolinium, may still be required as they are more sensitive for identifying CSF leaks compared to magnetic resonance imaging. However, these techniques require highly skilled operators, have a high radiation exposure, and are not widely available [1, 12].

Treatment of spinal CSF leak brings additional challenges. Conservative treatments (e.g., bed rest, hydration) and non-targeted epidural blood patch can still be initiated without neuroimaging evidence of spinal CSF leak, ideally within 2 weeks from symptom onset [11]. However, patients often wait longer than this to be seen by a specialist, thereby delaying treatment [13]. Conservative treatments are also unsuccessful in the majority of individuals, whilst epidural blood patch is effective in only 64% of individuals [1]. More invasive treatments (e.g., targeted blood patch and surgery) require neuroimaging evidence of the leak site and highly experienced practitioners [11]. However, surgical intervention does not guarantee resolution of the condition, whilst those treated after approximately 3 months of symptom onset are more likely to experience ongoing headaches [14]. This underscores the importance of early diagnosis and treatment in the management of spinal CSF leak [11].

In addition to diagnostic and treatment challenges, the overall patient experience of spinal CSF leak is burdensome. Previous studies have identified significant impacts on employment and financial well-being [13], and notable rates of depression, anxiety, and stress [15]. Despite quality-of-life research gaining increasing global attention over the past 5 years [6], the literature describing the lived experience of CSF leak is confined to Europe and North America, where healthcare systems may differ from other high-income countries. Furthermore, very few studies have recruited individuals from outside of tertiary care centres or institutions, resulting in likely underrepresentation of individuals with potentially less severe symptoms or more favourable treatment responses.

To address these gaps, this study aimed to map the socio-demographics of individuals with lived experience of spinal CSF leak across Australia and Aotearoa New Zealand (NZ), their range of symptoms, the diagnostic and treatment pathway, and the impact of their current symptoms on quality of life to better understand the healthcare challenges and unmet needs.

## Methods

### Ethics approval and informed consent

The study, including all participant-facing study materials, was approved by The University of Melbourne Human Research Ethics Committee (Project ID 31496) and adhered to the tenets of the Declaration of Helsinki. Participation was voluntary; participants were provided with a written Plain Language Statement and confirmed their informed consent prior to participation in the survey.

### Participants

Participants were required to be aged 18 years or older, proficient in the English language, and self-reported receiving a formal diagnosis and treatment for spinal CSF leak. Participants reporting exclusively cranial (or skull-based) CSF leak were excluded, as symptomatology is inherently different from spinal CSF leak (typically CSF rhinorrhoea, CSF otorrhea, and salty or metallic taste with no orthostatic headache) [16, 17].

### Survey design

A cross-sectional online survey was co-developed in collaboration with a consumer advisor with lived experience of spinal CSF leak (RLS; Online Resource 1) and reported in accordance with the Checklist for Reporting Results of Internet E-Surveys (Table S1, Online Resource 2) [18]. To constrain recall to a specific timepoint (as the condition could be ongoing), the survey evaluated the lived experience of an individual’s “first” CSF leak, however that was personally conceptualised (e.g., an isolated experience, a treated but recurrent experience, or an ongoing experience). Questions were formulated based on prior literature [13, 19–23] or generated de novo, and included 5 main sections: participant demographics, symptomatology, diagnostic pathway, treatment, and current impact of symptoms on quality of life. There was a maximum of 58 questions distributed over 32 webpages, with each page having 1–6 questions. Participants could not return to previous pages to review their answers. Participants could pause and resume the survey at any time; however, once the survey was started, participants had a maximum of 2 weeks to complete the survey. Upon survey completion, participants could opt-in to receive an e-giftcard of value $50 Australian dollars.

Survey branching was used, where responses were required to proceed and participants were only shown sections relevant to their earlier responses. Response options were in nominal, ordinal, interval, or open-ended formats. The final section of the survey was only available to respondents reporting current CSF leak symptoms. This component included the Australian or NZ version (as relevant to the participant’s reported country of residence) of the standardised EuroQol 5-Dimension 5-Level instrument (EQ-5D-5L) health-related quality of life measure [24, 25] and Headache Impact Test-6 (HIT-6) [26]. The EQ-5D-5L comprises Likert scale questions covering 5 dimensions (mobility, self-care, usual activities, pain/discomfort, and anxiety/depression) and the EuroQol Visual Analogue Scale (EQ-VAS), which is a self-reported rating system for general health against a scale of 0 (“worst health you can imagine”) to 100 (“best health you can imagine”) [24, 25]. Permission to use the EQ-5D-5L and EQ-VAS under a non-commercial use agreement was granted from the EuroQol Research Foundation (Registration #69420, Approval Date: 14 December 2024). The HIT-6 questionnaire is a 6-item short-form survey that measures the impact of headache [26]. This tool is widely used by clinicians and, therefore, familiar to people with CSF leak to describe and communicate how they feel and what they cannot do because of their headaches [27].

The survey was pretested independently by 3 researchers with extensive survey development experience (LSWK, ACBJ, BNN) and 2 people with lived experience of spinal CSF leak outside the authorship team to ensure readability, comprehension, face validity, and functionality and to determine the anticipated time commitment to complete the survey (~ 60 min).

### Survey dissemination

Electronic survey responses were collected from May to August 2025 via Qualtrics^®^ (Provo, Utah, United States). Convenience sampling was used due to the rarity of spinal CSF leak. The study was advertised electronically primarily through the CSF Leakers DownUnder Facebook patient support group (overseen by multiple administrators to ensure that content pertaining to local information, experiences, and support was safe and respectful) and Spinal CSF Leak Australia not-for-profit organisation (dedicated to supporting those with spinal CSF leak and raising awareness of the condition). In addition, electronic and hardcopy advertisements were sent to clinician networks (including neuroradiologists, neurosurgeons, and neurologists) and disseminated by word of mouth. Participants self-identified to participate in the study based on the eligibility requirements and did not require a password to enter the survey. None were approached by investigators directly.

### Data analysis

To achieve a margin of error of 10% at a confidence level of 95%, and assuming a predominant recruitment source from the CSF Leakers DownUnder Facebook support group population (~ 1600 members as of December 2024), we set an a priori desired sample size of 91 survey responses. Raw response data were stored on a secure SharePoint server managed by The University of Melbourne and only accessible by two of the study investigators (LSWK, BNN). Only completed surveys were included in the analysis and missing data were not computed. Several methods were implemented to triage for potentially unreliable or fraudulent survey responses [28], including manual review of individual responses (LSWK, BNN), identification of duplicate IP addresses, flagging of survey completion in a geolocation outside of Australia or NZ, and analysis of proprietary Qualtrics^®^ metrics for duplicate detection [29]. Where duplicate responses were identified, only the most recent entry was included in the analysis.

EQ-5D-5L utility scores were derived using Australian [30] and NZ [31] value sets dependent on the participant's country of residence. EQ-VAS scores were reported out of 100. Response scores and interpretation of headache severity impact for the HIT-6 were derived as per the HIT-6 User’s Manual (< 49: little-to-no impact; 50–55: some impact; 56–59: substantial impact; 60: severe impact) [27].

Qualitative data from open-text responses were pooled from across the whole survey for inductive reflexive thematic analysis [32]. One member with experience in qualitative research developed a coding framework (LSWK) using QSR NVivo version 15 (QSR International Pty Ltd, Melbourne, Australia), based on initial familiarisation with the text-based data, followed by initial grouping and organising of themes and sub-themes. Themes were discussed in an iterative process with a consumer advisor (RLS) and co-investigators with qualitative research expertise (ACBJ, BNN) to develop a refined representation of the CSF leak experience. The final thematic grouping was confirmed by 4 authors (LSWK, RLS, ACBJ, BNN) as part of the research triangulation strategy. Given different backgrounds, identities, and experiences of the authorship team involved in the qualitative data analysis, positionality statements are provided in Table S2 (Online Resource 2) to acknowledge the inevitability of bias in shaping our perspectives.

### Statistical analysis

Statistical analysis of quantitative data was performed using SPSS version 30.0 for Windows (IBM/SPSS Inc., Chicago, IL, USA). Data normality was assessed using the Shapiro–Wilk test. Continuous variables were presented as mean [± standard deviation (SD)] or median (interquartile range [IQR]) for normally or non-normally distributed data, respectively. Categorical data were presented as counts and percentages.

The Kruskal–Wallis test was applied to nonparametric ordinal variables for comparing the rate of difficulty-based Likert responses with time intervals taken between seeing a healthcare professional and obtaining a diagnosis and between obtaining a diagnosis and receiving treatment. The Wilcoxon Signed-Rank Test was used for paired samples to compare the amount of upright time between a self-perceived “good day” and “bad day.” Univariate and multivariate logistic regression was performed to evaluate predictors associated with the level of difficulty obtaining a diagnosis and obtaining treatment for the “first” spinal CSF leak, and to evaluate predictors associated with being currently symptomatic. Difficulty-based Likert scale responses were combined into bivariate outcomes; i.e., "very difficult" and "somewhat difficult" were merged into a "difficult" group, and "very easy" and "somewhat easy" were merged into an "easy" group for logistic regression modelling, excluding neutral responses. Univariate and multivariate linear regression was performed to evaluate predictors associated with EQ-VAS and HIT-6 scores. Predictor variables included in the analyses, where relevant, were: age at diagnosis (< 40 or ≥ 40 years), sex assigned at birth (female or male), time elapsed between relevant events (< 3 or ≥ 3 months between consulting a healthcare professional to diagnosis, and < 1 or ≥ 1 month from diagnosis to treatment), and symptom experienced. Variables that showed significance at *p* < 0.1 in the univariate analysis were included in a multivariate regression analysis using a backward stepwise approach (*p* < 0.05).

## Results

### Demographics of survey participants

A total of 106 participants finished the survey, with a completion rate of 65.0% (106/163 individuals who consented to participate). Their demographic information is summarised in Table 1. Most participants were aged 40–59 years (65/106, 61.3%), whilst 89/106 (84.0%) were female, 60/106 (56.6%) had obtained a Bachelor’s degree or higher, 59/106 (55.7%) were unemployed, and 71/106 (67.0%) were either married or in a de facto relationship. More than half of the participants were diagnosed with a spinal CSF leak between 30 and 49 years of age (55/106, 51.9%). At the time of survey completion, most participants reported being diagnosed with spinal CSF leak within the last 10 years (59/106, 55.7%).

**Table 1:** Socio-demographic characteristics of survey participants (*N* = 106)

Characteristic	*n* (%)
Country of residence	
Australia	97 (91.5)
NZ	9 (8.5)
Female sex at birth	89 (84.0)
Age at survey completion, years	
18–24	4 (3.8)
25–29	6 (5.7)
30–39	20 (18.9)
40–49	31 (29.2)
50–59	34 (32.1)
≥ 60	11 (10.4)
Age at first spinal CSF leak, years	
< 18	4 (3.8)
18–24	3 (2.8)
25–29	11 (10.4)
30–39	28 (26.4)
40–49	27 (25.5)
50–59	24 (22.6)
≥ 60	9 (8.5)
Highest level of education obtained	
Primary school	1 (0.9)
Secondary school	10 (9.4)
Certificate I or II	2 (1.9)
Certificate III or IV	12 (11.3)
Advanced diploma or diploma	19 (17.9)
Bachelor’s degree	29 (27.4)
Graduate diploma or graduate certificate	11 (10.4)
Postgraduate degree	20 (18.9)
Prefer not to say	2 (1.9)
Current employment^a^	
Unemployed	59 (55.7)
Volunteer	4 (3.8)
Home duties	5 (4.7)
Retired	9 (8.5)
Unable to work	42 (39.6)
Received income support or work cover payments	16 (15.1)
Student	5 (4.7)
marital status	
Never married	21 (19.8)
Married or de facto	71 (67.0)
Divorced/separated/widowed	14 (13.2)

*NZ* Aotearoa New Zealand, *CSF* cerebrospinal fluid

^a^Percentage sum exceeds 100.0% as participants could select more than one option as relevant to their situation

### Thematic analysis of open-ended text responses

**Fig. 1:**
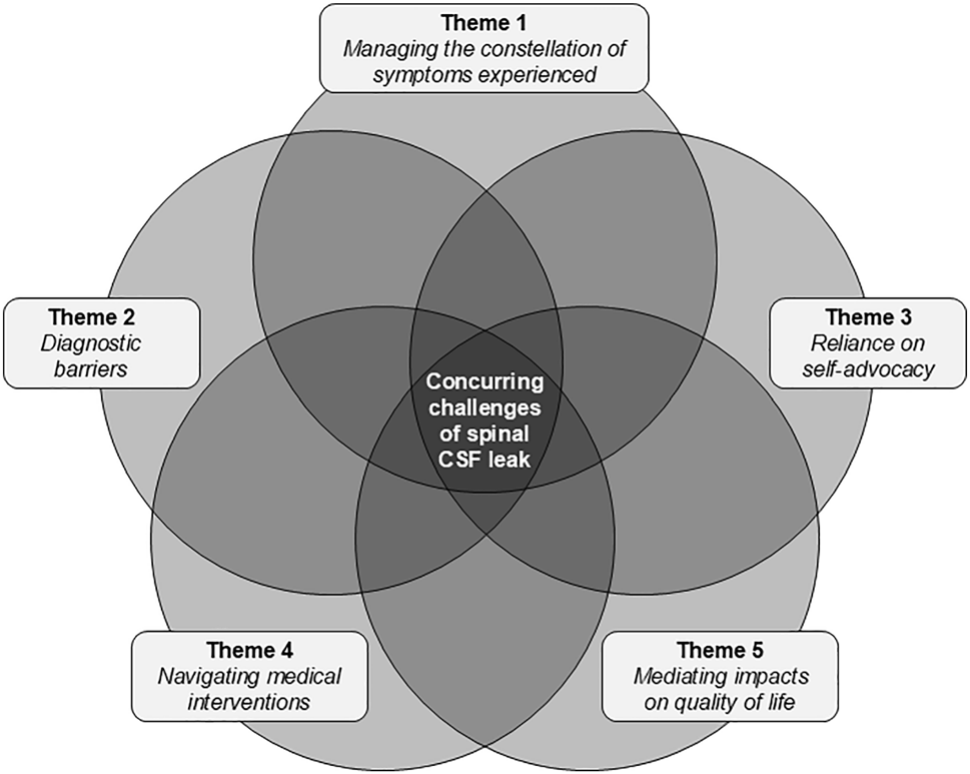
Visual representation of the 5 key themes (challenges) identified from inductive thematic analysis of all open-ended survey responses. Each challenge may occur on its own or in combination with other challenges, thereby compounding the difficulties of living with a spinal CSF leak

The results of the thematic analysis can be summarised under 5 major themes (Fig. 1): (1) managing the constellation of symptoms experienced, (2) diagnostic barriers, (3) reliance on self-advocacy, (4) navigating medical interventions, and (5) mediating impacts on quality of life. These themes represent the perceived and compounding challenges of the patient experience of their “first” spinal CSF leak, evident within the open-text survey responses. Four of the 5 themes mapped to the pre-defined survey sections: symptomatology, diagnostic pathway, treatment, and current impact of symptoms on quality of life. As such, both quantitative and thematic data are reported under each respective section, including supporting quotes to provide context and corroborate with the quantitative data. Descriptions of sub-themes and further supporting quotes are provided in Table S3 in Online Resource 2.

#### Experience of CSF leak symptoms

**Fig. 2:**
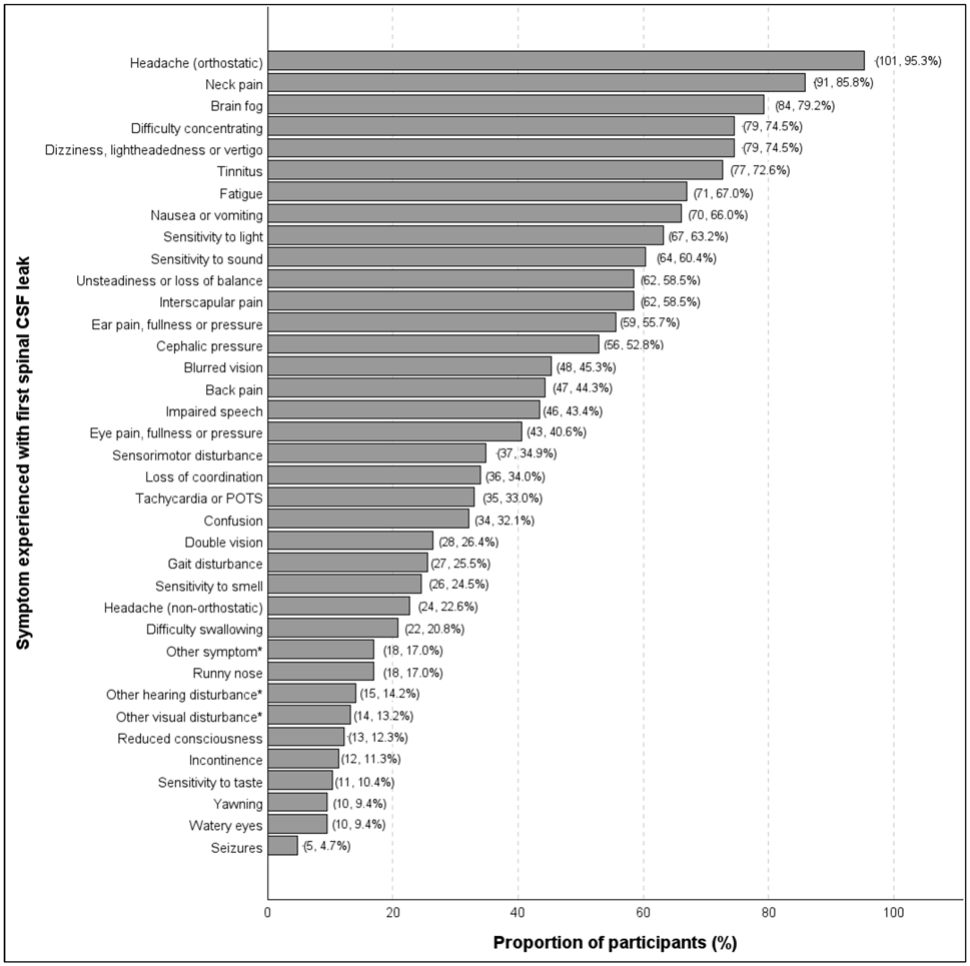
Nature and prevalence of symptoms experienced as part of the “first” CSF leak, reported by 106 survey participants. *Frequency values denote the number of individuals who reported additional symptoms under the categories of “other hearing disturbances”, “other visual disturbances”, and “other symptoms.” Verbatim responses for these categories are reported in Table S4 in Online Resource 2. POTS = postural orthostatic tachycardia syndrome

Participants reported more than 70 symptoms of their “first” CSF leak (Fig. 2). Overall, the most frequent symptoms reported were orthostatic headache (101/106, 95.3%), neck pain (91/106, 85.8%), and brain fog (84/106, 79.2%) (Fig. 2). Participants could also provide a free-text response to indicate additional symptoms not captured by the fixed response options, which included: hearing disturbances other than tinnitus, sensitivity to sound, ear pain, fullness, or pressure (e.g., hearing loss, whooshing sounds); visual disturbances other than sensitivity to light, blurred vision, double vision, watery eyes, eye pain, fullness, or pressure (e.g., visual snow, visual migraine); and “other symptoms” in general, many of which are related to a disorder of the parasympathetic nervous system (e.g., gastroparesis, increased perspiration). For a detailed summary of all the clinical features and symptoms (broadly characterised into neurological, ocular, vestibulocochlear, musculoskeletal, and “other” symptoms) as reported by survey participants, including verbatim quotes, see Table S4 (Online Resource 2).

Within the first theme of ‘Managing the constellation of symptoms experienced’ (Fig. 1), participants highlighted the intensity of the breadth of symptoms associated with their “first” spinal CSF leak. Orthostatic headache was frequently described as a unique headache experience, whilst other auditory, visual, and cognitive disturbances were often considered debilitating (see Table 2 for representative quotes about the severity of symptoms). Participants also reported the most helpful self-initiated strategies to mitigate their symptoms, such as recumbency, increased caffeine intake, increased hydration, and ice packs (i.e., conservative managements). Some participants mentioned that the use of opioids, sedatives, and migraine medication were required to lessen symptoms, subject to prescription from a medical practitioner, while others were unable to obtain any relief regardless of the strategy used.

**Table 2:** Representative participant quotes describing severity of symptoms experienced

Symptom	Participant quote
Orthostatic headache	"*The [orthostatic] headache was the most challenging. It consumed me. No normal life was able with the headache*.” (P59)
Cognitive disturbances	"*The most debilitating of all would be the cognitive symptoms – brain fog, memory problems, difficulty communicating. It felt like I could not access a lot of my mind…. It made me feel so isolated, and trapped inside a body that would not work*.” (P105)
Hearing disturbances	*“[The tinnitus] is unrelenting noise torture. There is no limit to the number of overlapping competing tones and the volume it can get. Some are lucky and only get it mild. Mine is catastrophic… [It] is literal profound torture*.” (P23)
Visual disturbances	“*The visual disturbances created a “vertigo” and were almost totally incapacitating. Together with nausea, the symptoms prevented me from engaging in normal activities*.” (P72)

#### Experience of CSF leak diagnostic pathway

Participants sought medical care from many different healthcare professionals to obtain a diagnosis of their “first” spinal CSF leak. Participants reported most commonly consulting neurologists (65/106, 61.3%), neurosurgeons (61/106, 57.5%), general practitioners (60/106, 56.6%), neuroradiologists, interventional radiologist or neuro-interventional radiologists (52/106, 49.1%), and hospital emergency doctors (51/106, 48.1%). Optometrists, ophthalmologists, and physiotherapists each had similar consultation rates (17/106, 16.0%) whilst ear, nose, and throat specialists and anaesthetists were each consulted less frequently (15/106, 14.2%). Various reasons for seeking healthcare for their “first” CSF leak were reported by participants, most commonly because their symptoms were severe (84/106, 79.2%), impacting on their life (80/106, 75.5%) or continuing to worsen (77/106, 72.6%).

**Fig. 3:**
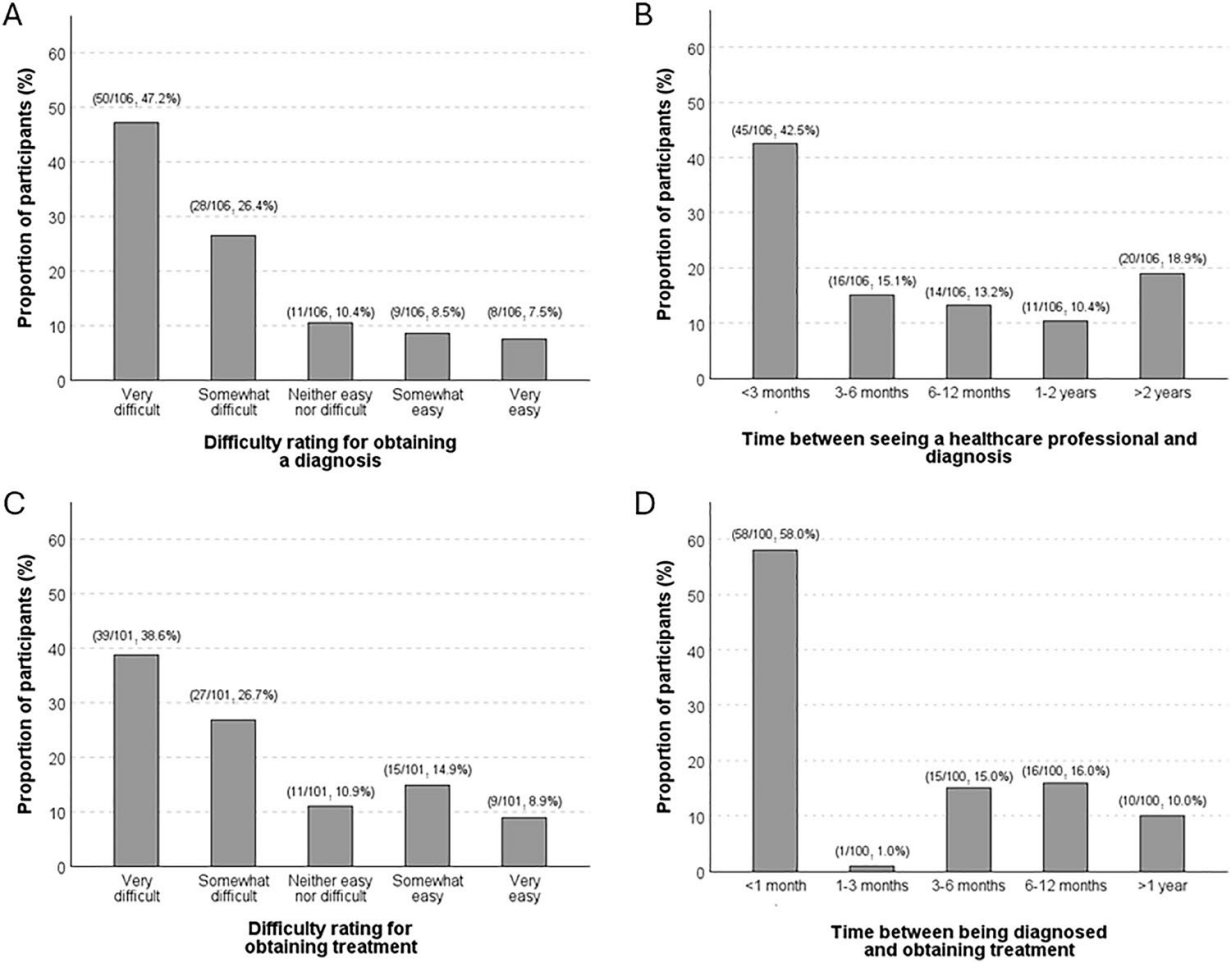
Diagnostic and treatment difficulty rating and timelines for a person’s “first” CSF leak**. A** Difficulty rating for obtaining a diagnosis (*N* = 106); **B** time between seeing a healthcare professional and obtaining a diagnosis (*N* = 106); **C** difficulty rating for obtaining treatment (*N* = 101); **D** time between being diagnosed and obtaining treatment amongst participants (*N* = 100)

The majority of participants considered that obtaining a diagnosis of their “first” spinal CSF leak was "very difficult" (50/106, 47.2%) or "somewhat difficult" (28/106, 26.4%), whilst few rated their experience as "somewhat easy" (9/106, 8.5%) or "very easy" (8/106, 7.5%) as per Fig. 3A. Less than half of participants were diagnosed within 3 months of seeing a healthcare professional (45/106, 42.5%; Fig. 3B).

There was a significant difference in participants’ difficulty ratings relative to the time elapsed between seeing a healthcare professional and obtaining a diagnosis (*p* < 0.001, Kruskal–Wallis test). After adjusting for other predictor variables, diagnosis of their “first” CSF leak within 3 months of seeing a healthcare professional was associated with a self-reported “easy” diagnosis experience (odds ratio [OR] 20.4; 95% confidence interval [CI] 4.3–96.7; *p* < 0.001; Table S1, Online Resource 3). Additional summary data regarding other health professionals consulted to obtain a diagnosis, other reasons for seeking healthcare, time elapsed between symptom onset and seeking healthcare, and self-reported procedures undergone to diagnose a CSF leak are provided in Table S5 (Online Resource 2).

Potential reasons why diagnosis of the “first” CSF leak was difficult were captured within the second qualitative theme of ‘Diagnostic barriers’ (Fig. 1). Participants reported limited clinician awareness and understanding of the condition. Many participants stated that they received alternate diagnoses (e.g., migraine, mental health issue, functional neurological disorder).

*“The most challenging part of the diagnostic process was the uncertainty and fear that came with not knowing what was wrong with me. I was repeatedly told I ‘just had migraines,’ despite knowing that something felt very different and more serious. I spent hours undergoing scans — MRIs, CTs, X-rays —whilst feeling increasingly anxious that something was being missed. The turning point came when I was finally told I had ‘many and multiple leaks.’”* (P95)

Specialist workforce shortage also contributed to difficulties obtaining a diagnosis. Many participants mentioned that it was costly to seek care from healthcare professionals with relevant experience in imaging, diagnosing, and treating spinal CSF leak, often having to travel interstate or internationally.

*“There needs to be more education for doctors in Australia as I went to so many specialists. But also there needs to be more specialists that can confidently read [digital subtraction myelography] imaging to get second opinions... It was extremely hard to get a second opinion and I ended up reaching out to specialists [overseas].”* (P34)

Difficulties in obtaining a diagnosis spurred many participants to rely on their ability to self-advocate, as captured in Theme 3, ‘Reliance on self-advocacy’ (Fig. 1). Most participants resorted to independently researching about CSF leaks to help understand their illness and symptom management, potential diagnostic and treatment procedures, and relevant health professionals. Information was obtained from various sources including online support groups, dedicated spinal CSF leak websites, and medical research articles.

*“The most helpful thing was having access through my university to medical journals, and the skills required, to do my own research. I studied clinical reports, original research, case reports, systematic reviews and meta-analyses to learn everything I could about CSF leaks, and ultimately spearhead my diagnosis pathway - without which, I could very well have been waiting decades for a solution.”* (P105)

Several participants discussed difficulties in developing perseverance and resilience as they sought appropriate healthcare, but this was made easier by social supports and having their symptoms validated by clinicians. Fewer participants expressed feelings of defeat and exhaustion when they sought a diagnosis and treatment.

*“There is a strong psychological battle that patients must face. They are forced to reach for spiritual faith; physical exhaustion takes hold and allies that show understanding and empathy is critical. I have no doubt suicidal thoughts become prevalent in CSF leakers.”* (P60)

#### Experience of treatment for spinal CSF leak

Participants’ treatment experiences were varied. At the time of survey completion, few participants had exclusively been managed conservatively with bed rest or hydration (14/106, 13.2%), whilst most participants reported having had at least one invasive medical procedure (92/106, 87.0%). The most common invasive procedure was an epidural blood patch (74/106, 69.8%), followed by surgical repair (33/106, 31.1%) and venous fistula embolisation (8/106, 7.5%), with one-quarter of respondents requiring more than one type of procedure (25/92, 27.2%).

Most participants perceived their experience of obtaining treatment for their “first” CSF leak to be very or somewhat difficult (66/101, 65.3%), compared to those who considered the process to be very or somewhat easy (24/101, 23.8%; Fig. 3C). Over half were treated within 1 month of diagnosis (58/100, 58.0%), but some participants (10/100, 10.0%) reported waiting more than 1 year before being treated after diagnosis (Fig. 3D). There was a significant difference in the treatment difficulty rating relative to the time elapsed between obtaining a diagnosis and receiving treatment (*p* < 0.001, Kruskal–Wallis test). Receiving treatment within 1 month of diagnosis was the only significant predictor variable associated with an “easy” treatment process (OR 5.2; 95%CI 1.6–16.8; *p* = 0.006; Table S2, Online Resource 3). For a full list of characteristics of CSF leak treatment reported by the survey participants, including types of treatment and time taken to obtain treatment, see Table S6 (Online Resource 2).

Many participants indicated challenges within the fourth theme of qualitative analysis: ‘Navigating medical interventions’ (Fig. 1). Barriers to treatment included treatment delays, limited access to treating specialists, high costs, and travel required.

*“By the time I finally got surgery, I'd been leaking for 21 months. Because of this, I still have residual symptoms and do not feel normal... Also the New Zealand healthcare system is so slow. It took three months after my myelogram to have the follow up appointment with the surgeon.”* (P25)

Conversely, knowledgeable and supportive healthcare professionals facilitated the treatment process. Participants often expressed their appreciation for having their spinal CSF leak experience validated and treatment plans communicated by healthcare professionals.

*“It made such a difference having a specialist who knew what they were talking about - someone I could trust. All the staff at the hospital - the nurses, doctors, general anaesthetists, orderlies etc. were lovely and very considerate.”* (P105)

Participants also frequently indicated that medical treatment outcomes were variable. Some experienced incomplete or no therapeutic benefit. Rebound intracranial hypertension was a commonly described post-operative complication.

*“I had significant improvement in upright time following my first two epidural blood patches. However, I experienced rebound intracranial hypertension following this and then my leak symptoms returned, however, they were less severe.”* (P86)

Post-operative care instructions were frequently considered to be lacking. Many participants described receiving no rehabilitative advice by their treating team, whilst others described conflicting advice between surgical and rehabilitative care teams.

*“Information provided to patients after surgery is lacking and sometimes was incorrect. I find this infuriating especially given the seriousness and difficulty of spinal surgeries. It adds to anxiety..."* (P97)

#### Current impact of CSF leak symptoms on quality of life

Over two-thirds of respondents were currently symptomatic (78/106, 73.6%). After adjusting for other predictor variables, diagnosis within 3 months of seeing a healthcare professional was associated with a decreased likelihood of being currently symptomatic (OR 0.4; 95%CI 0.1–0.9; *p* = 0.03; Table S3, Online Resource 3). Amongst those still experiencing symptoms, the majority experienced ‘headache and other symptoms’ (61/78, 78.2%), whilst reports of experiencing ongoing ‘headache only’ (1/78, 1.3%) and ‘symptoms other than headache’ (16/78, 20.5%) were less frequent (Table 3).

**Table 3:** Impact of current CSF leak symptoms, as reported by survey participants

Characteristic	
Profile of all participants (*N* = 106)	
Nature of current symptoms, *n* (%)	
Headaches only	1 (0.9)
Symptoms other than headaches	16 (15.1)
Headaches and other symptoms	61 (57.5)
Not experiencing symptoms	28 (26.4)
Profile of symptomatic patients (*N* = 78)	
Comfortableness on a “good day”, *n* (%)	
Not at all comfortable	10 (12.8)
Not very comfortable	36 (46.2)
Neutral	7 (9.0)
Somewhat comfortable	19 (24.4)
Very comfortable	6 (7.7)
Comfortableness on a “bad day”, *n* (%)	
Not at all comfortable	48 (61.5)
Not very comfortable	23 (29.5)
Neutral	3 (3.8)
Somewhat comfortable	2 (2.6)
Very comfortable	2 (2.6)
Hours of upright time, median (IQR)	
Hours of upright time on a “good day”	6 (3–10)
Hours of upright time on a “bad day”	2 (0.5–5)
EQ-5D-5L quality of life questionnaire, median (IQR)	
EQ-VAS score	40 (25–64)
EuroQol utility index	0.54 (0.26–0.80)
Profile of participants reporting current headache (*N* = 62)	
Total HIT-6 headache impact questionnaire score, mean ± SD	69 ± 5
Severity of headache impact, *n* (%)	
Little or no impact (score: ≤ 49)	0 (0.0)
Some impact (score: 50–55)	1 (1.6)
Substantial impact (score: 56–59)	1 (1.6)
Severe impact (score ≥ 60)	60 (96.8)

*N* number of participants who responded to the question, *IQR* interquartile range, *EQ-5D-5L* EuroQol 5-Dimension 5-Level instrument, *EQ-VAS* EuroQol Visual Analogue Scale, *HIT-6* Headache Impact Test-6, *SD* standard deviation

The comfort ratings of symptomatic participants are reported in Table 3. On an average “good day”, almost one-third of participants (25/78, 32.1%) considered themselves to be very or somewhat comfortable. On a “bad day”, very few participants considered themselves to be very (2/78, 2.6%) or somewhat comfortable (2/78, 2.6%). Median upright time on a “good day” was significantly higher than median upright time on a “bad day” (6 hours [IQR 3–10 hours] vs 2 hours [IQR 0.5–5 hours], respectively, *p* < 0.001, Wilcoxon Signed-Rank Test).

**Fig. 4:**
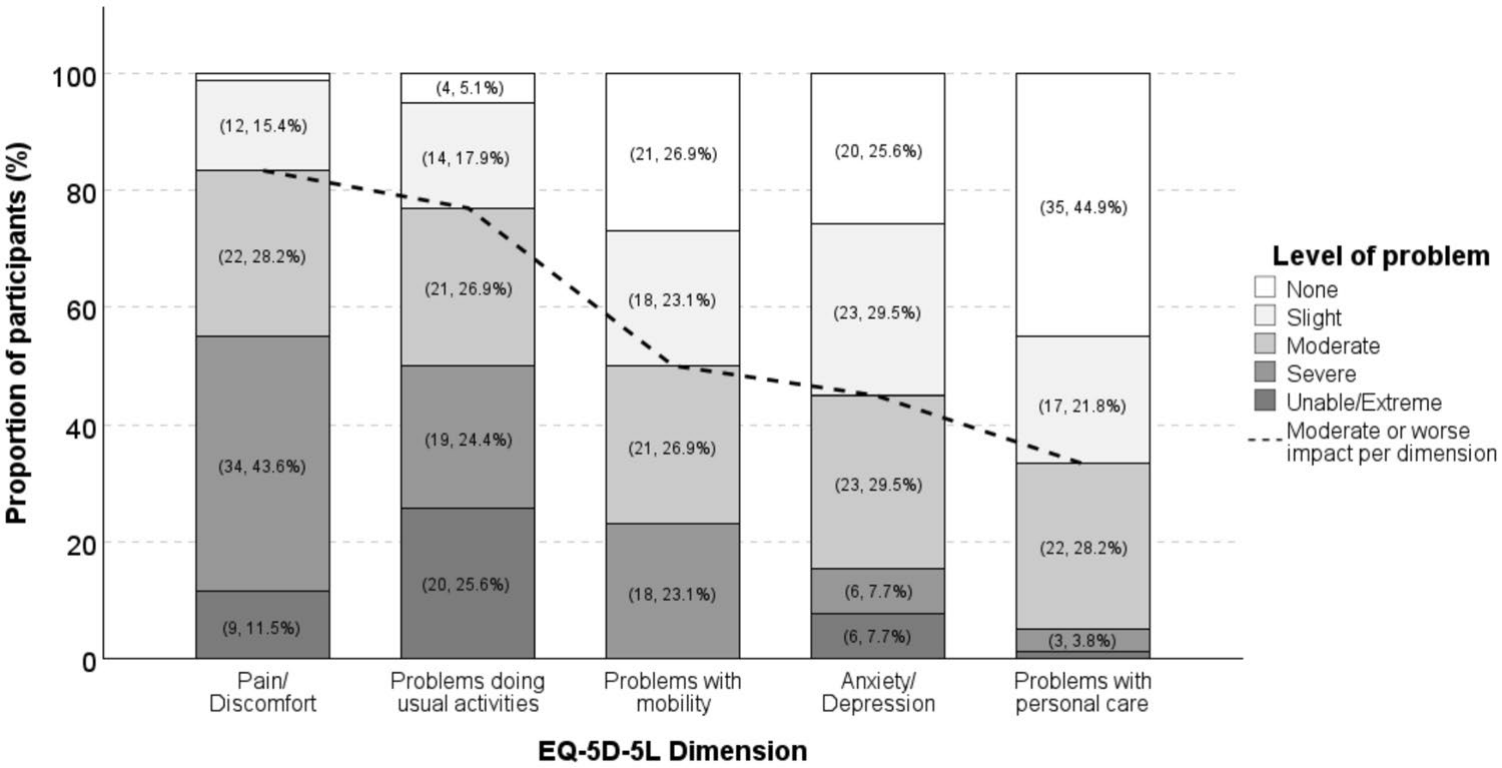
Participant ratings for the level of problem per EQ-5D-5L dimension (*N* = 78). Data values are presented as (*n*, %). The following values were not labelled for brevity: pain/discomfort (no problems [1, 1.3%]), problems with personal care (unable to do/extreme [1, 1.3%]), and mobility (unable [0, 0.0%])

The overall EQ-5D-5L median utility score was 0.54 (IQR 0.26–0.80) and median EQ-VAS score was 40 (IQR 25–64). In a multivariate linear regression model, currently symptomatic participants were significantly more likely to record a lower EQ-VAS score if they were diagnosed before age 40 years (unstandardised coefficient *B* = -12; 95%CI -22 to -2, *p* = 0.02; Table S4, Online Resource 3) or experienced headache (unstandardised coefficient *B* = -25; 95%CI -37 to -13, *p* < 0.001; Table S4, Online Resource 3). Participants more frequently reported a moderate or worse impact across EQ-5D-5L dimensions including pain or discomfort (65/78, 83.3%) and usual activities (60/78, 76.9%) compared to mobility (39/78, 50.0%), anxiety or depression (35/78, 44.9%), and personal care (26/78, 33.3%), as depicted in Fig. 4.

The HIT-6 was completed by 62 participants who reported currently experiencing headache symptoms, with a mean score of 69 (± 5 SD; Table 3). Univariate linear regression demonstrated that there were no significant predictors for HIT-6 scores, with participants diagnosed before age 40 years scoring similar to those diagnosed after 40 years of age (unstandardised coefficient *B* = 2; 95%CI -1 to 4, *p* = 0.21; Table S5, Online Resource 3). The majority of participants indicated that their headaches very often or always caused severe pain (48/62, 77.4%), limited daily activities (52/62, 83.9%), or made them want to lie down (56/62, 90.3%). When specifically asked about the past 4 weeks, most participants reported their headache very often or always made them feel irritated (50/62, 80.6%), too tired to perform work or daily activities (50/62, 80.6%), or limited their ability to concentrate (54/62, 87.1%). Consequently, almost all participants who were currently experiencing headache had cumulative HIT-6 scores indicating that their headache had a severe impact (score ≥ 60; 60/62, 96.8%; Table 3). For a complete summary of all HIT-6 results per dimension, see Table S7 (Online Resource 2).

Participants discussed many other factors that influenced their quality of life, which was captured by the final theme, ‘Mediating impacts on quality of life’ (Theme 5; Fig. 1). This included changes to social identity, whereby participants described changes in social connectedness. Participants often felt isolated by their condition and misunderstood, particularly as CSF leak is rare and public awareness is limited.

*“It is a very alienating disease, I didn't know anyone else with it. Most, including me, have never heard of it and even now that I can tell all my doctors in [City], they all admit that they don't know anything about it.”* (P83)

Participants also reported that their CSF leak imposed limitations on their work and parenting roles. Participating in usual hobbies or interests was challenging, particularly if frequently bed-bound.

*“It’s wrecked my life. I can’t be the wife, mother and grandmother I once was. No travel, no hobbies, little social interaction. It’s very isolating. It’s been nearly 6 years of headaches and surgeries that haven’t really helped.”* (P11)

Few participants reported that disruptions to quality of life were mediated by support from family, friends, and online support groups.

*“The support of CSF Leakers Downunder [has been most helpful]. Hearing the experiences of others helped me to be informed and feel less afraid. It normalised my experience.”* (P01)

Despite these challenges, some participants were able to adapt to their current level of function. This involved use of conservative managements (e.g., ice packs, periods of lying down), avoiding strenuous activity, and establishing routines to maximise their periods of being able to stay upright.

*“I have had to come to terms with a level of self-care that I was completely unpractised at. I have had to navigate this illness through research. Yet ultimately, I have learnt to pay attention to what my brain is telling me and as the symptoms can shift…quickly, sometimes I am unsure what I need to do to help myself.”* (P100)

Some participants further reported uncertainty of the future, including concerns of their condition regressing, and the possibility of not making a full recovery.

*“I worry about a recurrence of debilitating symptoms and also any long-term impacts on brain function.”* (P21)

## Discussion

This study provides a novel account of the lived experiences and patient journeys in an Australian and NZ cohort with spinal CSF leak, a population that has not been extensively featured in the CSF leak literature to date. Importantly, consumer involvement was key to all aspects of the study, including conception, design, analysis, and interpretation of results, ensuring relevance, quality, and impact of findings and tangible benefits to the CSF leak community. Overall, we found similar results and themes in the accounts of CSF leak patients of their symptomatology, diagnosis, treatment, and impact on quality of life reported in other parts of the world, highlighting the widespread challenges of debilitating symptoms, elusive diagnosis and treatment, and need for self-advocacy.

Whilst orthostatic headache is considered the hallmark symptom of spinal CSF leak, many other neurological, musculoskeletal, vestibular, and visual symptoms have been reported [11]. In this Australian and NZ cohort, and a recent cohort from North America [33], the relative frequencies of symptoms for an individual’s CSF leak, other than orthostatic headache, were notably higher compared to previous reports [1, 5, 20]. Over 70% of participants in this study, and the cohort study from North America [33], reported neck pain, brain fog, difficulty concentrating, dizziness, light-headedness or vertigo, and tinnitus. In comparison, a recent meta-analysis of spontaneous intracranial hypotension symptoms found that neck pain was estimated to occur in almost half of individuals with spinal CSF leak, tinnitus and dizziness in less than one-third of individuals, and cognitive symptoms in only 6% of individuals [1]. Similarly, the specific visual symptom of “blurred vision” was reported more frequently by this cohort (45%) and the United States cohort (66%)[33] compared to 14% of those who experienced any combination of blurred vision, nystagmus, and/or vision loss reported in the previous meta-analysis [1]. It may be that our participants were more likely to report a certain symptom, because they were directly prompted [23, 33], given the large list of potential symptoms to choose from in this study (see survey questions and options in Online Resource 1) and the inventory of questions used in the North American survey study [33]. It is also possible that those who participated in this study had a spinal CSF leak more recently, such that symptoms were easier to recall or their intensity was more noticeable; however, this could not be accurately ascertained as only an estimated half of participants completed the survey within 10 years of diagnosis. Symptom prevalence and intensity can be reduced within 3 months of onset [20], possibly due to compensatory CSF fluid dynamics [34]. Nonetheless, such diverse symptomology of spinal CSF leak reported here and elsewhere [1, 5, 20] has important implications for clinical diagnosis. Healthcare professionals should not only be aware of the characteristic orthostatic headache but actively enquire about a range of additional symptoms to improve spinal CSF leak diagnostic rates [11].

Timely diagnosis of spinal CSF leak requires coordinated efforts between multiple healthcare professionals [11]. As expected, neurologists were the most common healthcare professionals consulted as reported by our surveyed cohort (61.3%). This is in keeping with recommendations from the United Kingdom for initial neurology referral with specialist review within 4 weeks for non-urgent situations and escalation to a multidisciplinary centre (comprising neurologists, neurosurgeons, and neuroradiologists) if required [11]. However, there are a limited number of multidisciplinary spinal CSF leak clinics in Australia and none in NZ, which may influence healthcare referral pathways and timing of care in these specific geographical contexts.

Despite the recommendations from the United Kingdom, more than half of the participants in this study waited longer than 3 months to be diagnosed, the critical period for which treatment and intervention can be most effective [14, 35]. This highlights the difficulty in obtaining a diagnosis, as seen in this study and others [13, 33, 36]. Our analysis of open-text responses in this survey provides some speculative reasons for the diagnostic delays, at least from a patient’s perspective, including specialist workforce shortage, unavailability of medical imaging, high costs, and inaccessible location of specialists. Another key contributing factor could be a lack of awareness of spinal CSF leak amongst frontline healthcare professionals as the ‘gatekeepers’ for appropriate referral to specialist care [37]. Without surveying clinicians directly, it is not possible from the present study to comment on health professional perspectives explicitly. Nevertheless, a survey study in the United Kingdom reported that less than 5% of primary care practitioners (i.e., general practitioners) and emergency medicine physicians were confident in recognising the symptoms of spontaneous intracranial hypotension [38]. This highlights the importance of understanding current practitioner awareness, knowledge and behaviours, and design educational interventions accordingly, as part of future efforts to improve diagnosis of spinal CSF leak.

Other than orthostatic headache, the sheer number and variability of non-specific symptoms of spinal CSF leak across multiple bodily systems likely contributes to the diagnostic difficulties. Previous studies have only reported engagement with primary care physicians, emergency specialists, neurologists, neuroradiologists, and neurosurgeons [13, 36], seldom investigating consultation rates for other health disciplines. This is despite complaints of visual, auditory, and musculoskeletal symptoms associated with spinal CSF leak, which may lead primary care practitioners to refer participants to ophthalmic (optometrists, ophthalmologists), auditory (ear, nose, and throat specialists), and movement and function specialists (physiotherapists), respectively, instead of referring participants directly to neurologists or neurosurgeons. One study reported 44.2% of individuals consulted a physiotherapist, although it is unclear whether this occurred before or after diagnosis of spinal CSF leak [21]. Ocular and vestibulocochlear symptoms rarely occur in isolation in spinal CSF leak (particularly without an orthostatic headache), with few case reports documenting this (e.g., diplopia in an abducens nerve palsy [39] or isolated hearing and balance deficits similar to Meniere’s disease) [40]. Nonetheless, we show, for the first time, that it is not uncommon for people with spinal CSF leak symptoms to consult health professionals in disciplines outside of neurology as part of their diagnostic journey. Accordingly, increased awareness and education of spinal CSF leak amongst a range of healthcare professionals, not only in neurology and related disciplines but also in general practice and allied health, could facilitate more timely referrals and diagnoses.

Obtaining medical treatment for spinal CSF leak in Australia and NZ for the “first” spinal CSF leak was reported to be challenging, which is not an isolated finding considering similar reports of treatment difficulties worldwide [13, 36]. Whilst time from symptom onset to treatment was not captured in this study, only 59% of participants indicated receiving treatment within 3 months of diagnosis. Meanwhile, those diagnosed within 3 months of seeing a healthcare professional were significantly more likely to be asymptomatic at the time of survey completion. Better clinical outcomes are reported in those diagnosed and treated within 12 weeks of symptom onset [14, 35], emphasising the importance of timely access to CSF leak care. Barriers to seeking medical treatment from the patient perspective were similar to those experienced when obtaining a diagnosis (e.g., access to treating specialists).

Treatment outcomes may have also been influenced by CSF leak subtype and exact timing, procedure type, and number of procedures [1], but this study was not designed to evaluate such information and would require analysis of medical records. Nevertheless, our open-text response analysis indicated two key treatment challenges: rebound hypertension post-treatment and lack of, or insufficient, post-operative care. Similar findings have been reported in Canada [36] and the United Kingdom [13]. Recent post-operative care recommendations have incorporated management guidelines for rebound hypertension and follow-up regimes to address these common challenges [11]. Future studies could evaluate the uptake, effectiveness, and feasibility of the ideal post-operative guidelines (taking into account the real-world experience of patients with varying access to family/carer support and financial capacity for ongoing healthcare) and assess their overall impact on patient outcomes.

We found that the impact of spinal CSF leak on quality of life is large, consistent with the previous studies [13, 22]. Individuals may experience severe headaches with other symptoms, whilst median upright time was 2 or 6 hours, dependent on whether the day was considered “bad” or “good”, respectively. Whilst open-text analysis demonstrated that some individuals can adapt to their current level of function, there are concerns for recurrent or worsening symptoms. Meanwhile, quantitative measures of quality of life should be interpreted with caution, particularly as the EQ-5D-5L is not a specific measure of quality of life in spinal CSF leak [23]. However, it does provide useful information about a person’s perceived health-related quality of life overall. Amongst currently symptomatic participants, the median EQ-VAS score reported was 40. This is a considerably lower quality of life score than mean (± SD) EQ-VAS scores in individuals living with multiple sclerosis in Australia (68.7 ± 21.3) [41] and NZ (69.4 ± 21.7) [42] and population norms (Australia: 73.2 ± 21.7; [43] NZ: 74.8 ± 18.0) [44]. This may be explained by the compounding effects of limited and variable “upright” time, substantial impacts on usual activities, high levels of pain or discomfort, and social impacts of the condition (e.g., changes to social identity, connectedness and role functioning), given that over half of our participants reported unemployment (55.7%).

Similar and substantial socioeconomic impacts have been reported in Germany, the United Kingdom, and globally [13, 19, 21, 33]. Interestingly, our sub-analysis showed that lower EQ-VAS scores were recorded in individuals diagnosed before age 40 years, suggesting that the impacts of spinal CSF leak are greater for younger compared to older adults. However, this requires further investigation relative to the social context of the population studied to better understand the complexity and range of factors underlying the potential relationship between quality of life with age. Younger age, however, was not associated with greater impact of headache in this study, because HIT-6 scores were substantially skewed. Almost all participants with ongoing headache reported a severe impact score (69 ± 5), comparable to studies of people with chronic migraine (66.2 ± 4.3) [45]. Taken together, these two standardised questionnaires (EQ-5D-5L and HIT-6) enables quantification of the impact of current CSF leak symptoms with easily interpretable scores and, therefore, could be implemented as part of a systematic protocol to track individual progression over time in both research and clinical settings.

This study was instigated in response to patient advocacy identifying a need for statistics and data that could be used to highlight the real-world experience of people living with spinal CSF leak in Australia and NZ instead of relying on estimates from other countries. From the outset, we worked in collaboration with a consumer advisory group, with representatives from the newly established (in October 2024) not-for-profit Spinal CSF Leak Australia organisation and longstanding social network support group (CSF Leakers DownUnder Facebook support group) for people in Australia and NZ. Accordingly, a large proportion of participant recruitment occurred through the CSF Leakers DownUnder patient support group. Whilst online surveys are accessible and enables a high-volume of data to be collected, we acknowledge the limitations inherent to an e-survey study design, namely, self-reported information that is retrospective and subject to recall bias. Whilst some members may join the online patient support group after many years of suffering, anecdotally, most active members are typically earlier on in their journey (e.g., with a recent diagnosis and receiving initial treatment) with more recent lived experience, mitigating the risk of recall bias. Moreover, clinical information was self-reported, and we were unable to confirm the diagnostic criteria and investigative and treatment procedures used by clinicians. Nevertheless, we acknowledge that our data, at best, represent self-reported information about spinal CSF leak and the experience of the condition from the patient perspective. Using this self-reported data as a scaffold, future studies would benefit from accessing individuals’ medical records with their consent to confirm exact diagnoses and other clinical details to enable more comprehensive analysis of leak type, imaging modalities, and treatment responses than was possible here.

We acknowledge that survey responses were also subject to selection bias, and respondents with certain characteristics may have been more likely to complete the survey (e.g., individuals with higher level of education, female sex, those who have a greater presence on social media, or those with enough cognitive reserve to complete a survey). Based on anecdotal reports and discussions with the moderators/administrators of the online support group, those participants recruited through the CSF Leakers DownUnder patient support group likely represent a more diverse cross-section of people with CSF leak in the community than those referred to a tertiary medical centre. Other studies have typically recruited participants from their pool of patients in tertiary institutions [22, 33] or speciality CSF leak centres, [5, 19, 20, 23] whilst fewer studies have recruited nationwide through spinal CSF leak foundations [13, 36]. Therefore, we consider that our sampled cohort likely provides a more varied, real-world representation of the lived experience of spinal CSF leak in the community in Australia and NZ.

This study has identified that the experience of spinal CSF leak in Australia and NZ, from symptom onset, diagnosis and treatment experience, and quality of life, is consistent with that reported in other high-income countries. The findings contribute to a growing evidence base for clinician and patient advocacy and support mechanisms, including efforts to raise awareness of the heterogeneity of spinal CSF leak and improve patient outcomes. Further international and collaborative efforts are required to increase global awareness of spinal CSF leak and support consistent and accessible referral, diagnostic, and treatment pathways. These findings can help inform education and training of clinicians, so that our healthcare workforce is best equipped to improve access to care, ensure more timely diagnoses and treatments, and mitigate the impact on those living with spinal CSF leak.

## Supplementary Information

Below is the link to the electronic supplementary material.

Supplementary file1 (PDF 270 KB)

Supplementary file2 (PDF 328 KB)

Supplementary file3 (PDF 258 KB)

## Data Availability

The datasets used and/or analysed during the current study are available from the corresponding author on reasonable request.

## Abbreviations

CSF: Cerebrospinal fluid
NZ: Aotearoa New Zealand
EQ-5D-5L: EuroQol 5-Dimension 5-Level instrument
EQ-VAS: EuroQol Visual Analogue Scale
HIT-6: Headache Impact Test-6
SD: Standard deviation
IQR: Interquartile range
OR: Odds ratio
CI: Confidence interval
