# Supplementary material for: “Getting to diagnosis was an absolute nightmare”: survey insights about the lived experience of spinal CSF leak in Australia and Aotearoa New Zealand
Source: J Neurol. 2026 Apr 30;273(5):300. doi: 10.1007/s00415-026-13840-y (PMC13132889; doi:10.1007/s00415-026-13840-y)
Supplement: Supplementary file 1 — Supplementary file1 (PDF 270 KB) [file 415_2026_13840_MOESM1_ESM.pdf]

Online Resource 1: e-Survey (Journal of Neurology)

"Getting to diagnosis was an absolute nightmare": survey insights about the lived experience of spinal CSF leak in Australia and Aotearoa New Zealand

Lachlan SW Knight,[a,b] Rachel L Smith,[a,c] Alexis Ceecee Britten-Jones,[a,d] Sam E John,[e,f] David B Grayden,[e] Bang V Bui,[a] Lauren N Ayton,[a,d,g] Bao N Nguyen[a]

Affiliations:
[a]Department of Optometry and Vision Sciences, The University of Melbourne, Parkville, Victoria, Australia
[b]Flinders University, College of Medicine and Public Health, Flinders Health and Medical Research Institute, Adelaide, South Australia, Australia
[c]Spinal CSF Leak Australia and CSF Leakers DownUnder patient support group, Australia
[d]Centre for Eye Research Australia, Royal Victorian Eye and Ear Hospital, Melbourne, Victoria, Australia
[e]Department of Biomedical Engineering and Graeme Clark Institute for Biomedical Engineering, The University of Melbourne, Parkville, Victoria, Australia
[f]Department of Medicine, The University of Melbourne, Parkville, Victoria, Australia
[g]Department of Surgery (Ophthalmology), The University of Melbourne, Parkville, Victoria, Australia

Corresponding author:
Dr Bao Nguyen, Department of Optometry and Vision Sciences, The University of Melbourne
Email: bnguyen@unimelb.edu.au Phone: +61 3 9035 8553

The survey was implemented in Qualtrics. The questions appear in fixed order, as per the blocks below.
*indicates response validation (mandatory question) or text box validation (mandatory answer in appropriate text or number form)

Block 0 – Introduction

ID	Question/Instructions	Options
0.0	Thank you for your interest in our survey about the lived experience of cerebrospinal fluid (CSF) leak in Australia and New Zealand. Please read the Plain Language Statement by clicking on the arrow to proceed.	
0.1	[Placeholder for digital version of Plain Language Statement]	
0.2	As this survey is anonymous, we are unable to provide you with a physical copy of the Plain Language Statement. However, you can download a copy of the Plain Language Statement here [link to be made available] for you to keep.<br><br>If you have any questions about the study after reading the Plain Language Statement, please contact Dr Bao Nguyen, Department of Optometry and Vision Sciences, The University of Melbourne on (03) 9035 8553 or email bnguyen@unimelb.edu.au<br><br>If you wish to participate in the survey, please read the Survey Consent Form by clicking on the arrow to proceed.	
0.3	[Placeholder for digital version of Survey Consent Form]	
0.4	As this survey is anonymous, we are unable to provide you with a physical copy of the Consent Form. However, you can download a copy of the Consent Form here [link to be made available] for you to keep.<br><br>Do you agree to the above statements and consent to participate in the survey? By clicking on the arrow to proceed, this record of consent will be retained by the researchers.	Yes, I do consent<br>No I do not consent#
0.5	Thank you for agreeing to complete the survey. Because your complete history is difficult to capture in a single survey or interview, the focus of this study is specifically constrained to the experience of your “first leak”, however you conceptualise that to be (i.e. the first and only leak ever experienced, a recurrent first leak, or the first of multiple leaks).<br><br>We value your participation in this survey, which will help us understand the lived experience of CSF leak. Your honest and thoughtful answers are crucial for the success of the research project.<br><br>Please answer all questions in order. Click >> to move forward, and << to move back. Please select one option only for multiple choice questions and use the text box to enter your answer in text form, unless otherwise indicated.<br><br>The survey is expected to take approximately 60 minutes, depending on your answers, and is broken up into 5 smaller sections. Not all sections are relevant to all participants and you can have a break at any time.<br>If you leave the survey at any point, you can resume progress later (within 2 weeks of beginning the survey) using the same browser on the same device. The survey will automatically start at the stage where you left it. If you wish to begin the survey now, please click >> to start.	

End survey if this option is selected

Block 1 – Demographic information

ID	Question/Instructions	Options
Header	**Part 1: Demographic information**	
1.0	The first questions are to confirm your eligibility to participate in the study, and helps us understand and characterise trends and patterns in the group of people we are surveying. This section is expected to take 2-3 minutes.	
1.1*	In which country do you currently live?	Australia<br>New Zealand<br>Other (please specify)*
1.2*	What is your current age?	< 18 years#<br>18-24 years<br>25-29 years<br>30-39 years<br>40-49 years<br>50-59 years<br>60 years or above
1.3*	How many leaks do you think you have had, to date?	1<br>2-3<br>4-5<br>6 or more
1.4*	How would you describe your first CSF leak?	Spinal CSF leak only<br>Cranial CSF leak only#<br>Both spinal and cranial CSF leak<br>Other (please specify)*
1.5*	In which country did you receive the diagnosis of your first CSF leak?<br>[Select all that apply]	Australia<br>New Zealand<br>Other (please specify)*<br>No formal diagnosis yet#
1.6*	How old were you when you received the diagnosis of your first CSF leak?	< 18 years<br>18-24 years<br>25-29 years<br>30-39 years<br>40-49 years<br>50-59 years<br>60 years or above
1.7*	In which country did you receive treatment for your first CSF leak?<br>[Select all that apply]	Australia<br>New Zealand<br>Other (please specify)*<br>No treatment yet#
1.8*	What was your sex recorded at birth?	Male<br>Female<br>Another term (please specify)*<br>Prefer not to say
1.9*	What is the highest level of education you have attained?	Primary school education<br>Secondary school education<br>Certificate I or II<br>Certificate III or IV<br>Advanced Diploma or Diploma<br>Bachelor Degree<br>Graduate Diploma or Graduate Certificate<br>Postgraduate Degree<br>Prefer not to say

1.10*	What is your current employment status?<br>[Select all that apply]	Employed<br>Student<br>Volunteer Work<br>Family or home duties<br>Retired<br>Unable to work<br>I receive income support payments<br>Other (please specify)*
1.11*	What is your current marital status?	Never married<br>De-facto<br>Married<br>Divorced<br>Separated<br>Widowed

End survey if this option is selected

Block 2 – Symptoms of your first CSF leak

ID	Question	Options
Header	**Part 2: Symptoms of your first CSF leak**	
2.0	Now that we understand a bit about you, we would like to ask about the symptoms of your first CSF leak prior to being diagnosed. Please think about the symptoms that you attribute to CSF leak, and not about symptoms from pre-existing conditions. Your input will help us further understand the range of symptoms people can experience with CSF leak, to know if we can improve diagnostic testing for CSF leak.<br><br>This part should take about 5-10 minutes to complete. If you wish to have a break and leave the survey at any point, you can resume progress later (within 2 weeks of beginning the survey) using the same browser on the same device. The survey will automatically start at the stage where you left it.	
2.1*	Which of the following symptoms did you experience with your first CSF leak? Please think about the symptoms that you attribute to CSF leak, and not about symptoms from pre-existing conditions. [Select all that apply]	Orthostatic headache (position-dependent headache that worsens when upright and is relieved with lying down)<br>Non-orthostatic headache (headache that does not change in severity with position)<br>Cephalic pressure ("pressure inside the skull")<br>Neck pain<br>Back pain<br>Interscapular pain (pain between the shoulder blades)<br>Ear pain, fullness or pressure<br>Eye pain, fullness or pressure<br>Sensitivity to light<br>Sensitivity to sound<br>Sensitivity to smell<br>Sensitivity to taste<br>Double vision (seeing two images of a single object)<br>Blurred vision

		Any other visual disturbance (please specify)*<br>Tinnitus (ringing in ears)<br>Hearing disturbance (other than tinnitus)<br>Sensorimotor disturbance (decreased sensations, diminished reflexes, muscle twitches, leg or arm weakness, numbness or paralysis)<br>Runny nose<br>Watery eyes<br>Nausea or vomiting<br>Dizziness, lightheadedness or vertigo (swaying or spinning sensation)<br>Unsteadiness or loss of balance<br>Lack of coordination<br>Gait disturbance (disrupted ability to walk)<br>Difficulty swallowing<br>Incontinence<br>Fatigue<br>Yawning<br>Tachycardia (fast heart beat) or POTS (postural orthostatic tachycardia syndrome)<br>Confusion<br>Impaired speech (difficulty finding words, difficulty forming words, stutter)<br>"Brain fog"<br>Difficulty concentrating<br>Reduced consciousness<br>Seizures<br>None of the above* [Go to Q2.2 if selected]
2.2*	What other symptoms (not mentioned already) did you experience with your first CSF leak? Please think about the symptoms that you attribute to CSF leak, and not about symptoms from pre-existing conditions.	[Open text box]
2.3*	Which symptom of your first CSF leak was the <u>most challenging</u> for you, and why?	[Open text box]
2.4*	What did you find <u>most helpful</u> for the symptoms of your first CSF leak, and why?	[Open text box]
2.5*	Is there anything else about your experience of <u>symptoms of your first CSF leak</u> that you would like to tell us? Your response can include feelings, thoughts, and suggestions for improvement (if any).	No<br>Yes*

__Block 3 – Diagnosis of your first CSF leak__

ID	Question	Options
Header	**Part 3: Diagnosis of your first CSF leak**	
3.0	Thank you for completing Parts 1 and 2 of the survey. The next set of questions will ask about your experience of diagnosis of your first CSF leak. Your input will help us further understand the healthcare pathways for people living with CSF leak, to know if we can improve investigations for CSF leak and health system utilisation.<br><br>This part should take about 15-20 minutes to complete. If you wish to have a break and leave the survey at any point, you can resume progress later (within 2 weeks of beginning the survey) using the same browser on the same device. The survey will automatically start at the stage where you left it.	
3.1*	Which health professional/s did you see as part of diagnosis of your first CSF leak?<br>[Select all that apply]	GP (general practitioner)<br>ED (hospital emergency department)<br>Urgent care clinic<br>Neurologist<br>Neuroradiologist, interventional radiologist or neuro-interventional radiologist<br>Neurosurgeon<br>Anaesthetist<br>Psychiatrist<br>Psychologist or psychotherapist<br>Optometrist<br>Ophthalmologist or Neuro-ophthalmologist<br>ENT (ear nose and throat) specialist<br>POTS (postural orthostatic tachycardia syndrome) specialist<br>Rheumatologist<br>Physiotherapist<br>Multidisciplinary CSF leak clinic<br>None of the above* [Go to Q3.2 if selected]<br>Unsure/I don't know
3.2*	What other health professionals (not mentioned already) did you see as part of diagnosis of your first CSF leak? To maintain anonymity, please indicate the profession when providing additional information, and not the real name of any person.	[Open text box]
3.3*	What initially prompted you to seek a professional opinion about your first CSF leak?<br>[Select all that apply]	I had multiple symptoms<br>My symptoms were severe<br>My symptoms were continuing and/or getting worse<br>My symptoms were impacting on my life<br>Someone who knows me personally (e.g. family member, friend, colleague) recommended me to seek a professional opinion<br>A health professional recommended me to seek another professional opinion (to maintain

		anonymity, please indicate the profession, and not the real name of any person, who gave you the recommendation)*<br>A support group recommended me to seek a professional opinion (please specify which support group gave the recommendation)*<br>I found information online (please specify which source of information/website)*<br>Other (please specify)*
3.4*	Approximately how much time passed between noticing the symptoms of your first CSF leak and presenting for a professional opinion, for the first time?	< 3 months<br>3-6 months<br>6-12 months<br>1-2 years<br>Other (please specify)*
3.5*	Which procedures did you undergo to diagnose your first CSF leak?<br>[Select all that apply]	MRI (magnetic resonance imaging) with contrast injection<br>MRI (magnetic resonance imaging) without contrast injection<br>MRM (magnetic resonance imaging myelogram)<br>CTM (computed tomography myelogram)<br>DSM (digital subtraction myelogram)<br>Lumbar puncture<br>Intracranial pressure monitoring (e.g. ICP bolt)<br>Cerebrospinal fluid flow monitoring (e.g. CSF scintigraphy)<br>Other surgery (please specify)*<br>None of the above [Go to Q3.6 if selected]<br>Unsure/I don't know
3.6*	What other procedures (not mentioned already) did you undergo as part of diagnosis of your first CSF leak?	[Open text box]
3.7*	Did your symptoms of your first CSF leak change after undergoing any diagnostic procedure/s? If so, how?	No<br>Yes*
3.8*	Approximately how much time passed between first presenting for a professional opinion and diagnosis of your first CSF leak?	< 3 months<br>3-6 months<br>6-12 months<br>1-2 years<br>Other (please specify)*
3.9*	Overall, how easy was it to get a diagnosis of your first CSF leak?<br>[Move the slider to indicate your selection]	[Visual scale ranging from "very difficult" at minimum 1 to "very easy" at maximum 5]
3.10*	What was the most challenging aspect of your experience of diagnosis of your first CSF leak, and why?	[Open text box]
3.11*	What did you find most helpful during your experience of diagnosis of your first CSF leak, and why?	[Open text box]
3.12*	Is there anything else about your experience of diagnosis of your first CSF leak that you would like to tell us? Your response can include feelings, thoughts, and suggestions for improvement (if any).	No<br>Yes*

Block 4 – Treatment for your first CSF leak

ID	Question	Options
Header	**Part 4: Treatment for your first CSF leak**	
4.0	Thank you for completing the survey questions so far; you are now approximately halfway.<br>The following Part 4 questions will ask about your experience of <u>treatment of your first CSF leak</u>. Your input will help us further understand the treatment pathways for people living with CSF leak, to know if we can improve the experience of treatment.<br><br>This part should take about 10-15 minutes to complete. If you wish to have a break and leave the survey at any point, you can resume progress later (within 2 weeks of beginning the survey) using the same browser on the same device. The survey will automatically start at the stage where you left it.	
4.1*	Which treatment/s did you receive for your first CSF leak?<br>[Select all that apply]	Conservative treatment (bed rest, hydration)<br>Blood patch without radiological guidance<br>Blood patch with radiological guidance<br>Venous fistula embolisation<br>Occipital nerve block<br>Caffeine infusion<br>Other surgery (please specify)*<br>Other medications (please specify)*<br>None of the above [Go to Q4.2 if selected]<br>Unsure/I don't know
4.2*	What other treatments (not mentioned already) did you receive for your first CSF leak?	[Open text box]
4.3*	Approximately how much time passed between diagnosis and treatment of your first CSF leak?	<1 month<br>1-3 months<br>3-6 months<br>6-12 months<br>Other (please specify)*
4.4*	Did your symptoms of your first CSF leak change after undergoing any treatment/s? If so, how?	No<br>Yes*
4.5*	Overall, how easy was it to get treatment of your first CSF leak?<br>[Move the slider to indicate your selection]	[Visual scale ranging from "very difficult" at minimum 1 to "very easy" at maximum 5]
4.6*	What was the <u>most challenging</u> aspect of your experience of treatment of your first CSF leak, and why?	[Open text box]
4.7*	What did you find <u>most helpful</u> during your experience of treatment of your first CSF leak, and why?	[Open text box]
4.8*	Is there anything else about your experience of <u>treatment of your first CSF leak</u> that you would like to tell us?<br><br>Your response can include feelings, thoughts, and suggestions for improvement (if any).	No<br>Yes*

Block 5 – Current impact of CSF leak symptoms

ID	Question	Options
Header	**Part 5: Impact of current CSF leak symptoms**	
5.0	Thank you for completing the survey questions so far. Part 5 is about the impact of current CSF leak symptoms. Some questions may not be relevant to everyone, depending on what symptoms you are currently experiencing.<br><br>Your input will help us further understand the impact of symptoms for people living with CSF leak, so that we can demonstrate the burden of living with CSF leak.<br><br>This part should take about 10-15 minutes to complete. If you wish to have a break and leave the survey at any point, you can resume progress later (within 2 weeks of beginning the survey) using the same browser on the same device. The survey will automatically start at the stage where you left it.	
5.1*	Do you currently have symptoms of CSF leak?	Yes, only headaches that are attributed to CSF leak<br>Yes, only symptoms other than headache that are attributed to CSF leak^<br>Yes, both headaches and other symptoms that are attributed to CSF leak<br>No [Go to Q9.0 if this is selected]
5.2*	Because symptoms can vary from day-to-day, we now ask you to think about your average "good day" (more positive) and "bad day" (more challenging) to describe the range of experience. **On a "good day"**, approximately how many hours in a day, in total, are you able to be upright?<br>[Please enter a number only]	[Text box for number entry]*
5.3*	**On a "good day"**, how comfortable are your current CSF leak symptoms?<br>[Move the slider to indicate your selection]	[Visual scale ranging from "not comfortable at all" at minimum 1 to "very comfortable" at maximum 5]
5.4*	**On a "bad day"**, approximately how many hours in a day, in total, are you able to be upright?<br>[Please enter a number only]	[Text box for number entry]*
5.5*	**On a "bad day"**, how comfortable are your current CSF leak symptoms?<br>[Move the slider to indicate your selection]	[Visual scale ranging from "not comfortable at all" at minimum 1 to "very comfortable" at maximum 5]
5.6*	What is the most challenging impact of your current CSF leak symptoms on your life, and why?	[Open text box]
5.7*	What do you find most helpful for your current CSF leak symptoms, and why?	[Open text box]
5.8*	Is there anything else about your experience of current CSF leak symptoms that you would like to tell us?<br>Your response can include feelings, thoughts, and suggestions for improvement (if any).	No<br>Yes*

^ Continue with rest of Section 5, 6 or 7 as appropriate, and Skip Section 8 if this option is selected

Block 6 – EQ-5D-5L Health Questionnaire (Australia)

ID	Question	Options
6.0	The next set of questions may look different to the rest of+ the survey. The questions come directly from an approved, international questionnaire to determine health-related quality of life.<br><br>Please follow the instructions on every page and click >> to continue.	[Appears only for those who choose Australia for Q1.1]
Header	**EQ-5D-5L Health Questionnaire (Australia)**	
6.1*	Mobility<br>[Please tap the ONE box that best describes your health TODAY]	I have no problems with walking around<br>I have slight problems with walking around<br>I have moderate problems with walking around<br>I have severe problems with walking around<br>I am unable to walk around
6.2*	Personal care<br>[Please tap the ONE box that best describes your health TODAY]	I have no problems with washing or dressing myself<br>I have slight problems with washing or dressing myself<br>I have moderate problems with washing or dressing myself<br>I have severe problems with washing or dressing myself<br>I am unable to wash or dress myself
6.3*	Usual activities (e.g. work, study, housework, family or leisure activities)<br>[Please tap the ONE box that best describes your health TODAY]	I have no problems doing my usual activities<br>I have slight problems doing my usual activities<br>I have moderate problems doing my usual activities<br>I have severe problems doing my usual activities<br>I am unable to do my usual activities
6.4*	Pain/discomfort<br>[Please tap the ONE box that best describes your health TODAY]	I have no pain or discomfort<br>I have slight pain or discomfort<br>I have moderate pain or discomfort<br>I have severe pain or discomfort<br>I have extreme pain or discomfort
6.5*	Anxiety/depression<br>[Please tap the ONE box that best describes your health TODAY]	I am not anxious or depressed<br>I am slightly anxious or depressed<br>I am moderately anxious or depressed<br>I am severely anxious or depressed<br>I am extremely anxious or depressed
6.6	We would like to know how good or bad your health is TODAY. This scale is numbered from 0 to 100. 100 means the BEST health you can imagine. 0 means the WORST health you can imagine.	
6.7*	Please tap on the scale to indicate how your health is TODAY.	[Visual scale of ticks, ranging from minimum 0 “worst health you can imagine” to maximum 100 “best health you can imagine”]

Block 7 – EQ-5D-5L Health Questionnaire (New Zealand)

**ID**	**Question**	**Options**
7.0	The next set of questions may look different to the rest of the survey. The questions come directly from an approved, international questionnaire to determine health-related quality of life.<br><br>Please follow the instructions on every page and click >> to continue.	[Appears only for those who choose New Zealand for Q1.1]
Header	**EQ-5D-5L Health Questionnaire (New Zealand)**	
7.1*	Mobility<br>[Please tap the ONE box that best describes your health TODAY]	I have no problems in walking about<br>I have slight problems in walking about<br>I have moderate problems in walking about<br>I have severe problems in walking about<br>I am unable to walk about
7.2*	Self-care<br>[Please tap the ONE box that best describes your health TODAY]	I have no problems washing or dressing myself<br>I have slight problems washing or dressing myself<br>I have moderate problems washing or dressing myself<br>I have severe problems washing or dressing myself<br>I am unable to wash or dress myself
7.3*	Usual activities (e.g. work, study, housework, family or leisure activities)<br>[Please tap the ONE box that best describes your health TODAY]	I have no problems doing my usual activities<br>I have slight problems doing my usual activities<br>I have moderate problems doing my usual activities<br>I have severe problems doing my usual activities<br>I am unable to do my usual activities
7.4*	Pain/discomfort<br>[Please tap the ONE box that best describes your health TODAY]	I have no pain or discomfort<br>I have slight pain or discomfort<br>I have moderate pain or discomfort<br>I have severe pain or discomfort<br>I have extreme pain or discomfort
7.5*	Anxiety/depression<br>[Please tap the ONE box that best describes your health TODAY]	I am not anxious or depressed<br>I am slightly anxious or depressed<br>I am moderately anxious or depressed<br>I am severely anxious or depressed<br>I am extremely anxious or depressed
7.6	We would like to know how good or bad your health is TODAY. This scale is numbered from 0 to 100. 100 means the BEST health you can imagine. 0 means the WORST health you can imagine.	
7.7*	Please tap on the scale to indicate how your health is TODAY.	[Visual scale of ticks, ranging from minimum 0 "worst health you can imagine" to maximum 100 "best health you can imagine"]

Block 8 – Headache Impact Test (HIT-6)

ID	Question	Options
8.0	The next set of questions come directly from an approved, clinical questionnaire to determine the impact of headaches.<br>Please click >> to continue.	[Appears only for those who choose headaches as part of current symptoms for Q5.1]
Header	**Headache Impact Test (HIT-6)**<br>This questionnaire was designed to help you describe and communicate the way you feel and what you cannot do because of headaches.	
8.1*	When you have headaches, how often is the pain severe?	Never<br>Rarely<br>Sometimes<br>Very often<br>Always
8.2*	How often do headaches limit your ability to do usual daily activities including household work, work, school, or social activities?	Never<br>Rarely<br>Sometimes<br>Very often<br>Always
8.3*	When you have a headache, how often do you wish you could lie down?	Never<br>Rarely<br>Sometimes<br>Very often<br>Always
8.4*	In the past 4 weeks, how often have you felt too tired to do work or daily activities because of your headaches?	Never<br>Rarely<br>Sometimes<br>Very often<br>Always
8.5*	In the past 4 weeks, how often have you felt fed up or irritated because of your headaches?	Never<br>Rarely<br>Sometimes<br>Very often<br>Always
8.6*	In the past 4 weeks, how often did headaches limit your ability to concentrate on work or daily activities?	Never<br>Rarely<br>Sometimes<br>Very often<br>Always

Block 9 – Contact details

ID	Question	Options
9.0	Thank you for your responses so far.<br>Below you can opt-in to provide your name and email address to our research team for future contact. You can choose if you would like to be contacted for one or more reasons. Providing your contact details will make your survey responses no longer anonymous.<br>After completing this section, you will see a confirmation message to say that all your survey responses have been recorded and you can exit the browser. Thank you!	

9.1*	I wish to:<br>• receive a $50 prepaid Mastercard giftcard. Please note that the voucher can only be activated by a valid email address and cannot be forwarded to another email address, so please indicate your best contact email address when prompted.<br>• receive a summary of the results of this study<br>• be contacted for a potential one-to-one interview about your experience with CSF leak (Phase 2 of this study)<br>• be contacted for other related studies in the future	[Drop down menu for each]<br><br>Yes [Go to Q9.2 if selected]<br>No#
9.2*	Please provide your name and email address	[Email address entry]*

End survey if this option is selected

End of survey message

Thank you for completing the survey. Your responses have been recorded.

If you have any questions about the study, please contact Dr Bao Nguyen, Department of Optometry and Vision Sciences, The University of Melbourne on (03) 9035 8553 or email bnguyen@unimelb.edu.au
