# Supplementary material for: “Getting to diagnosis was an absolute nightmare”: survey insights about the lived experience of spinal CSF leak in Australia and Aotearoa New Zealand
Source: J Neurol. 2026 Apr 30;273(5):300. doi: 10.1007/s00415-026-13840-y (PMC13132889; doi:10.1007/s00415-026-13840-y)
Supplement: Supplementary file 2 — Supplementary file2 (PDF 328 KB) [file 415_2026_13840_MOESM2_ESM.pdf]

Online Resource 2: Supplementary Information (Journal of Neurology)

"Getting to diagnosis was an absolute nightmare": survey insights about the lived experience of spinal CSF leak in Australia and Aotearoa New Zealand

Lachlan SW Knight,[a,b] Rachel L Smith,[a,c] Alexis Ceecee Britten-Jones,[a,d] Sam E John,[e,f] David B Grayden,[e] Bang V Bui,[a] Lauren N Ayton,[a,d,g] Bao N Nguyen[a]

Affiliations:
[a]Department of Optometry and Vision Sciences, The University of Melbourne, Parkville, Victoria, Australia
[b]Flinders University, College of Medicine and Public Health, Flinders Health and Medical Research Institute, Adelaide, South Australia, Australia
[c]Spinal CSF Leak Australia and CSF Leakers DownUnder patient support group, Australia
[d]Centre for Eye Research Australia, Royal Victorian Eye and Ear Hospital, Melbourne, Victoria, Australia
[e]Department of Biomedical Engineering and Graeme Clark Institute for Biomedical Engineering, The University of Melbourne, Parkville, Victoria, Australia
[f]Department of Medicine, The University of Melbourne, Parkville, Victoria, Australia
[g]Department of Surgery (Ophthalmology), The University of Melbourne, Parkville, Victoria, Australia

Corresponding author:
Dr Bao Nguyen, Department of Optometry and Vision Sciences, The University of Melbourne
Email: bnguyen@unimelb.edu.au Phone: +61 3 9035 8553

Table S1. Checklist for Reporting Results of Internet E-Surveys

Item Category	Checklist Item	Explanation
**Design**	Target population	Individuals aged ≥18 years with a self-reported formal diagnosis of spinal CSF leak and had received treatment for spinal CSF leak.
	Sampling	Convenience sampling was used due to spinal CSF leak being a rare condition.
**Institutional Review Board (IRB) approval and informed consent process**	IRB approval	This study was approved by the University of Melbourne Human Research Ethics Committee (Project ID: 31496).
	Informed consent	Prior to entering the survey, participants were provided with a consent form and were required to provide consent before proceeding.
	Data protection	Digital data was stored in the University of Melbourne password protected Qualtrics and SharePoint servers.
**Development and pre-testing**	Development and testing	The survey was developed by the authors based on literature review and input from a consumer with lived experience of spinal CSF leak. It was pretested independently by researchers to ensure readability and functionality.
**Recruitment process and description of the sample having access to the survey**	Open survey versus closed survey	The survey was an open survey and did not require a password to enter.
	Contact mode	Researchers did not have initial contact with any potential participant. Instead, the study was advertised as per below.
	Advertising the survey	The survey was advertised through several means. Firstly, it was advertised through the CSF Leakers DownUnder Facebook support group, which is an online, closed patient support group. It was also advertised on the Spinal CSF Leak Australia website. Furthermore, an advertisement and letter of invitation was sent by email or hardcopy to clinicians identified through relevant clinician networks, where clinicians could place the advertisement on a noticeboard or similar, or give the advertisement to patients of whom may be interested in participating. Finally, the study was advertised via word of mouth for snowball recruitment.
**Survey administration**	Web/E-mail	The survey was administered online via Qualtrics, where responses were automatically stored.
	Context	The survey was posted online on specific websites developed to support individuals with CSF leak (CSF Leakers DownUnder closed Facebook support group and Spinal CSF Leak Australia). CSF Leakers DownUnder is overseen by three administrators/advocates to ensure that content pertaining to local information, experiences and support is safe and respectful. Spinal CSF Leak Australia is a charity organisation dedicated to supporting those with spinal CSF leak and raising awareness of the condition.

	Mandatory/ voluntary	Participation in the survey was voluntary.
	Incentives	Participants who completed the survey had the option to opt-in to receive an e-giftcard with a value of $50 Australian dollars if their contact details were provided.
	Time/Date	The survey was open from May to August 2025
	Randomisation of items or surveys	Items were not randomised or alternated. Instead, the questions were presented in sections with a logical flow.
	Adaptive questioning	Survey branching was used, and sections were only presented to participants based on their previous responses.
	Number of items	The number of items per page varied from 1 to 6.
	Number of screens	The survey was distributed over 32 pages with up to 58 questions.
	Completeness check	Completeness checks were done during the survey as a response was required for all questions before proceeding to the next.
	Review step	Participants were not able to review their answers via a Back button.
**Response rates**	Unique site visitor	IP addresses were used to determine unique site visitor alongside Qualtrics metrics to determine duplicate responses.
	View rate	n/a
	Participation rate	n/a
	Completion rate	Completion rate was 65.0% (106/163 individuals who consented to participate).
**Preventing multiple entries from the same individual**	Cookies used	Qualtrics browser cookies, to prevent multiple sessions, were used.
	IP Check	IP addresses were used to identify potential duplicate responses from the same user. In the event duplicate complete entries occurred, the most recent response was kept for analysis.
	Log file analysis	Qualtrics® system quality checks to detect duplicates were used.
	Registration	n/a
**Analysis**	Handling of incomplete surveys	Only completed surveys were analysed.
	Surveys submitted with an atypical time stamp	A pre-specified completion timeframe cut-off point was not used. Individual responses were instead screened to ensure valid responses were recorded and analysed.
	Statistical correction	n/a

n/a: not applicable

Table S2. Researcher positionality statements for qualitative analysis

Author	Statement
Lachlan SW Knight	Cis-gender male clinician-scientist, with a European Australian background, working in rare ocular and neurological disease. His interest in spinal CSF leak is shaped by his commitment to helping those who often experience systemic disadvantage and limited visibility within the healthcare system.
Rachel L Smith	A consumer advisor with lived experience of spinal CSF leak in Australia. Shaped by a personal commitment to improving access to healthcare and the quality of life for individuals impacted by spinal CSF leak, she acknowledges and respects the diverse range of experiences of the condition across the wider Australian and New Zealand CSF leak community.
Alexis Ceecee Britten-Jones	Cis-gender female clinician-scientist from a multicultural background (first-generation New Zealander who migrated to Australia), with a foundation in vision and genetics research, particularly focusing on rare diseases. Her perspective is shaped by working closely with individuals affected by under-recognised and complex health conditions, with a particular interest in promoting integrated healthcare that is equitable and systematic.
Bao N Nguyen	Cis-gender female vision and neuroscience researcher, clinician, and educator with a culturally and linguistically diverse background (second generation Australian). Her interest in spinal CSF leak is motivated by her professional and clinical expertise in neurological conditions that affect the eye and visual system, and a passion for addressing inequalities in healthcare access.

Table S3. Major themes and subthemes summarising qualitative data

Major theme	Subtheme	Supporting quote
**1. Managing the constellation of symptoms experienced**	Constellation of symptoms	*"The symptoms were stressful and debilitating… Hearing distortions, vomiting, extreme fatigue, headaches, stiffness in neck. Symptoms went on for many months.*" (P84)
	Adopting conservative management	*"My symptoms improved from the forced bedrest and high level of caffeine I was given in that first week."* (P05)
	Escalating medical management	*"If I can't manage pain levels at home, I have gone to the emergency [department] three times and have had pain management there."* (P35)
**2. Diagnostic barriers**	Limited awareness and understanding of condition	*"Getting to diagnosis was an absolute nightmare… Overall a significant lack of awareness of the condition, its possible causes, and diagnostics put me at a real disadvantage and delayed treatment."* (P07)
	Specialist workforce and resource shortage	*"The challenging part was I live in [State], so every time [the specialist] ordered a diagnostic test, I needed to pay for flights, accommodation, plus testing costs. There are no CSF experts in [State], so going to [City] seemed like my best chance of getting an accurate diagnosis."* (P26)
**3. Reliance of self-advocacy**	Researching condition	*"Google knew more about CSF leaks than most medical professionals. I have done so much of my own research."* (P08)
	Developing perseverance	*"I was still leaking after so many months. I didn't give up fighting for my life back."* (P46)
**4. Navigating medical interventions**	Factors impacting choice and access	*"After diagnosis, [there was] no clarity about treatment options, and long delays to even get initially seen in the public system with near zero feedback about expected wait times. Things quickly started moving with an interstate private hospital."* (P36)
	Intervention outcome variability	*"Blood patches had no effect. Bed rest helped in the short term. Surgical repair happened twice… but 16 months post-op, symptoms changed again."* (P27)
**5. Mediating impacts on quality of life**	Navigating changes to social identity	*"There were so many symptoms that had a significant impact on every aspect of my life... I wasn't able to portray who I was on the inside, so it was a major loss of identity."* (P105)
	Adapting to current symptoms	*"I have a routine of doing things in the first half of the day. After midday I have no energy and the headaches roll in… It's been 6 years, so routine helps."* (P76)
	Managing uncertainty	*"I never know what a bad day will be. I can't exercise like I want to. I'm terrified I'll go back to being in as much pain as I was when my symptoms started."* (P08)

Table S4. Clinical features and reported symptoms of "first" CSF leak, as reported by survey participants (N=106). Abbreviations: CSF = cerebrospinal fluid; POTS = postural orthostatic tachycardia syndrome.

**Clinical feature**	**n (%)**
**Number of CSF leaks**	
1	64 (60.4)
2	20 (18.9)
≥3	22 (20.8)
**Type of CSF leak**	
Spinal CSF leak only	82 (77.4)
Spinal and cranial CSF leak	10 (9.4)
Spinal CSF-venous fistula	8 (7.5)
Spinal CSF-lymphatic fistula	6 (5.7)
**Country of diagnosis**	
Australia	97 (91.5)
New Zealand	9 (8.5)
**Reported symptoms**	
Neurological and cognitive symptoms	
Orthostatic headache	101 (95.3)
Brain fog	84 (79.2)
Difficulty concentrating	79 (74.5)
Dizziness, lightheadedness or vertigo	79 (74.5)
Fatigue	71 (67.0)
Nausea or vomiting	70 (66.0)
Unsteadiness or loss of balance	62 (58.5)
Cephalic pressure	56 (52.8)
Impaired speech	46 (43.4)
Sensorimotor disturbance	37 (34.9)
Loss of coordination	36 (34.0)
Tachycardia or POTS	35 (33.0)
Confusion	34 (32.1)
Gait disturbance	27 (25.5)
Sensitivity to smell	26 (24.5)
Non-orthostatic headache	24 (22.6)
Difficulty swallowing	22 (20.8)
Runny nose	18 (17.0)
Reduced consciousness	13 (12.3)
Incontinence	12 (11.3)
Sensitivity to taste	11 (10.4)
Yawning	10 (9.4)
Seizures	5 (4.7)

Musculoskeletal symptoms	
Neck pain	91 (85.8)
Interscapular pain	62 (58.5)
Back pain	47 (44.3)
Ocular symptoms	
Sensitivity to light	67 (63.2)
Blurred vision	48 (45.3)
Eye pain, fullness or pressure	43 (40.6)
Double vision	28 (26.4)
Watery eyes	10 (9.4)
Other visual disturbance (verbatim)<br>1. *"Visual snow and ghosting"*<br>2. *"Visual snow"*<br>3. *"Flashing lights, snow etc."*<br>4. *"Palinopsia, static/snow"*<br>5. *"Oscillopsia, palinopsia, flashing lights"*<br>6. *"Strobing and kaleidoscope visual effects"*<br>7. *"Ocular aura"*<br>8. *"A few occasional visual migraines"*<br>9. *"Vision loss"*<br>10. *"Distorted vision"*<br>11. *"Vision has to catch up head movements"*<br>12. *"Strabismus"*<br>13. *"Bright spot in right eye and floaters"*<br>14. *"Spots in vision"*	14 (13.2)
Vestibulocochlear symptoms	
Tinnitus (ringing in ears)	77 (72.6)
Sensitivity to sound	64 (60.4)
Ear pain, fullness or pressure	59 (55.7)
Other hearing disturbance (verbatim)<br>1. *"Unilateral neuro sensory hearing loss"*<br>2. *"Deafness in both ears. Highs in one, lows in the other"*<br>3. *"Hearing loss"*<br>4. *"Hearing loss"*<br>5. *"Echoed hearing, hearing loss/muffled hearing, chronic forms of tinnitus – buzzing/electrical type currents"*<br>6. *"Pulsatile tinnitus – I could hear my heartbeat in my head"*<br>7. *"Pulsatile tinnitus"*<br>8. *"Popping ears, fluttering sound in ears"*<br>9. *"Feeling of block ears, fluttering sound in one ear, loud droning/humming"*<br>10. *"Muffled sounds as though I'm underwater, whooshing sounds appearing to come from a different direction"*<br>11. *"Muffled hearing"*<br>12. *"Hearing muffled"*<br>13. *"Hearing vibrations"*<br>14. *"Hearing my eye movements, hearing voices/music in fans"*<br>15. *"A sound similar to an ultrasound especially when lying down"*	15 (14.2)

Other symptoms (verbatim)<br>1. *"Upper arm pain"*<br>2. *"Pressure across nasal region"*<br>3. *"Postnasal drip (continuous stream). Fluid collecting in small pools in my ears that would sometimes crystallise and/or cause sores"*<br>4. *"Poor temperature regulation, poor circulation inc. Raynaud's, myoclonic jerks, pain at base of skull / head feeling very heavy, memory problems, urinary retention, hormonal – acne and changes to menstrual cycle, increased sweating, worsening of pre-existing [irritable bowel syndrome] symptoms, difficulty with fine motor skills, loss of appetite, jaw pain"*<br>5. *"Perspiration"*<br>6. *"My headache never fully relieved when laying flat. Reduced slightly, l had severe neck spasms and had difficulty when laying flat to find a head position that eased symptoms slightly (i.e., head pain and spasms). Also had severe neck spasms when coughing or using bowels, passing wing or over reaching, or looking up"*<br>7. *"Lack of yawning"*<br>8. *"Jarring in top of head when walking, pain on coughing"*<br>9. *"Hand tremors"*<br>10. *"Formication (severe)"*<br>11. *"Feeling like a great weight on head dragging you downwards when trying to walk; low [intracranial pressure] on [intracranial pressure] monitoring machine"*<br>12. *"Fainting"*<br>13. *"Fainting"*<br>14. *"Extreme insomnia"*<br>15. *"Dystonia"*<br>16. *"Difficult holding up head sustainably"*<br>17. *"Bradycardia, low respiratory rate, facial pain and numbness, gastroparesis, constipation"*<br>18. *"Angry, domineering, metallic taste in mouth"*	18 (17.0)

Table S5. Characteristics of the CSF leak diagnostic pathway, as reported by survey participants. Abbreviations: N = number of participants who responded to the question; POTS = postural orthostatic tachycardia syndrome; CSF = cerebrospinal fluid; ICP = intracranial pressure

**Characteristic**	**n (%)**
**Health professional seen[a] (N=106)**	
Neurologist	65 (61.3)
Neurosurgeon	61 (57.5)
General practitioner	60 (56.6)
Neuroradiologist, interventional radiologist or neuro-interventional radiologist	52 (49.1)
Emergency doctor	51 (48.1)
Optometrist	17 (16.0)
Ophthalmologist or neuro-ophthalmologist	17 (16.0)
Physiotherapist	17 (16.0)
Anaesthetist	15 (14.2)
Ear, nose and throat specialist	15 (14.2)
Multidisciplinary CSF clinic	11 (10.4)
POTS specialist	9 (8.5)
Psychologist or psychotherapist	9 (8.5)
Rheumatologist	8 (7.5)
Psychiatrist	7 (6.6)
Urgent care	5 (4.7)
Other	9 (8.5)
Unsure/can't recall who I saw	0 (0.0)
**Initial prompt to seek health professional [a] (N=106)**	
Severe symptoms	84 (79.2)
Symptoms impacting life	80 (75.5)
Symptoms continuing to worsen	77 (72.6)
Multiple symptoms	64 (60.4)
Recommendation from healthcare professional	28 (26.4)
Recommendation from personal relationship	10 (9.4)
Found online information	20 (18.9)
Recommendation from support group	19 (17.9)
Other	4 (3.8)
**Time between symptom onset and seeking healthcare (N=106)**	
<3 months	77 (72.6)
3—6 months	7 (6.6)
6—12 months	7 (6.6)
1—2 years	8 (7.5)
>2 years	4 (3.8)
Unspecified	3 (2.8)

**Self-reported procedures undergone to diagnosis CSF leak [a] (N=106)**	
Magnetic resonance imaging	97 (91.5)
Computed tomography myelography	54 (50.9)
Lumbar puncture	38 (35.8)
Magnetic resonance imaging myelography	26 (24.5)
Digital subtraction myelography	23 (21.7)
ICP monitoring	11 (10.4)
CSF flow monitoring	3 (2.8)
Other	13 (12.3)
Unsure/can't recall what procedures I underwent	2 (1.9)
Not yet undergone any procedure for diagnosis of CSF leak	2 (1.9)
**Change in symptoms following investigations (N=103)**	
Yes	44 (42.7)
No	59 (57.3)
**Time between seeing a health professional and diagnosis (N=106)**	
<3 months	45 (42.5)
3—6 months	16 (15.1)
6—12 months	14 (13.2)
1—2 years	11 (10.4)
>2 years	20 (18.9)
**Ease of obtaining diagnosis for first spinal CSF leak (N=106)**	
Very easy	8 (7.5)
Somewhat easy	9 (8.5)
Neither easy nor difficult	11 (10.4)
Somewhat difficult	28 (26.4)
Very difficult	50 (47.2)

[a]Percentage sum exceeds 100.0% as participants could select more than one option as relevant to their situation.

Table S6. Characteristics of CSF leak treatment, as reported by survey participants. Abbreviations: N = number of participants who responded to the question; CSF = cerebrospinal fluid.

**Characteristic**	**n (%)**
**Location of treatment (N=106)**	
Australia or New Zealand only	103 (97.2)
Multiple countries	3 (2.8)
**Treatment received for first spinal CSF leak[a] (N=106)**	
Conservative treatment (bed rest, hydration)	60 (56.6)
Epidural blood patch	74 (69.8)
Venous fistula embolisation	8 (7.5)
Occipital nerve block	12 (11.3)
Caffeine infusion	4 (3.8)
Other surgery	33 (31.1)
Other medication	4 (3.8)
Cannot recall	0 (0.0)
**Time between diagnosis and treatment (N=100)**	
<1 month	58 (58.0)
1—3 months	1 (1.0)
3—6 months	15 (15.0)
6—12 months	16 (16.0)
>12 months	10 (10.0)
**Change in symptoms after treatment (N=101)**	
Yes	82 (81.2)
No	19 (18.8)
**Ease of obtaining treatment for first spinal CSF leak (N=101)**	
Very easy	9 (8.9)
Somewhat easy	15 (14.9)
Neither easy nor difficult	11 (10.9)
Somewhat difficult	27 (26.7)
Very difficult	39 (38.6)

[a]Percentage sum exceeds 100.0% as participants could select more than one option as relevant to their situation.

Table S7. HIT-6 questionnaire scores per dimension for the participants reporting current headache symptoms of CSF leak (N=62). Abbreviations: HIT = Headache Impact Test.

	Experience severe pain	Limit daily activities	Wish to lie down	Felt too tired	Felt fed up or irritated	Limited ability to concentrate
Never, n (%)	0 (0.0)	0 (0.0)	0 (0.0)	0 (0.0)	0 (0.0)	0 (0.0)
Rarely, n (%)	2 (3.2)	1 (1.6)	0 (0.0)	2 (3.2)	3 (4.8)	0 (0.0)
Sometimes, n (%)	12 (19.4)	9 (14.5)	6 (9.7)	10 (16.1)	9 (14.5)	8 (12.9)
Very often, n (%)	37 (59.7)	33 (53.2)	17 (27.4)	30 (48.4)	31 (50.0)	40 (64.5)
Always, n (%)	11 (17.7)	19 (30.6)	39 (62.9)	20 (32.3)	19 (30.6)	14 (22.6)
