# Supplementary material for: “Getting to diagnosis was an absolute nightmare”: survey insights about the lived experience of spinal CSF leak in Australia and Aotearoa New Zealand
Source: J Neurol. 2026 Apr 30;273(5):300. doi: 10.1007/s00415-026-13840-y (PMC13132889; doi:10.1007/s00415-026-13840-y)
Supplement: Supplementary file 3 — Supplementary file3 (PDF 258 KB) [file 415_2026_13840_MOESM3_ESM.pdf]

Online Resource 3: Regression Analyses (Journal of Neurology)

"Getting to diagnosis was an absolute nightmare": survey insights about the lived experience of spinal CSF leak in Australia and Aotearoa New Zealand

Lachlan SW Knight,[a,b] Rachel L Smith,[a,c] Alexis Ceecee Britten-Jones,[a,d] Sam E John,[e,f] David B Grayden,[e] Bang V Bui,[a] Lauren N Ayton,[a,d,g] Bao N Nguyen[a]

Affiliations:
[a]Department of Optometry and Vision Sciences, The University of Melbourne, Parkville, Victoria, Australia
[b]Flinders University, College of Medicine and Public Health, Flinders Health and Medical Research Institute, Adelaide, South Australia, Australia
[c]Spinal CSF Leak Australia and CSF Leakers DownUnder patient support group, Australia
[d]Centre for Eye Research Australia, Royal Victorian Eye and Ear Hospital, Melbourne, Victoria, Australia
[e]Department of Biomedical Engineering and Graeme Clark Institute for Biomedical Engineering, The University of Melbourne, Parkville, Victoria, Australia
[f]Department of Medicine, The University of Melbourne, Parkville, Victoria, Australia
[g]Department of Surgery (Ophthalmology), The University of Melbourne, Parkville, Victoria, Australia

Corresponding author:
Dr Bao Nguyen, Department of Optometry and Vision Sciences, The University of Melbourne
Email: bnguyen@unimelb.edu.au Phone: +61 3 9035 8553

Table S1. Univariate and multivariate backward stepwise logistic regression analysis assessing predictors for rating obtaining a diagnosis of a participant's "first" spinal CSF leak as easy

Covariate	N	Univariate logistic regression		Multivariate logistic regression Model 1		Multivariate logistic regression Model 4	
		**OR (95% CI)**	**P value**	**OR (95% CI)**	**P value**	**OR (95% CI)**	**P value**
**Sex**							
Male	16	1.0					
Female	79	1.6 (0.3—8.0)	0.54				
**Age at diagnosis**							
≥40 years	54	1.0					
<40 years	41	1.2 (0.4—3.5)	0.72				
**Time between seeing a healthcare professional and being diagnosed**							
≥3months	59	1.0					
<3 months	36	20.4 (4.3—96.7)	**<0.001**	17.9 (3.6—88.3)	**<0.001**	20.4 (4.3—96.7)	**<0.001**
**Orthostatic headache**							
No	5	1.0					
Yes	90	n/a	1.0				
**Non-orthostatic headache**							
No	74	1.0					
Yes	21	0.4 (0.1—2.0)	0.27				
**Brain fog**							
No	19	1.0					
Yes	76	0.8 (0.2—2.7)	0.69				
**Difficulty concentrating**							
No	24	1.0					
Yes	71	0.8 (0.2—2.5)	0.67				
**Dizziness, lightheadedness, or vertigo**							
No	24	1.0					
Yes	71	0.6 (0.2—1.7)	0.30				
**Fatigue**							
No	32	1.0					
Yes	63	0.5 (0.2—1.5)	0.20				

**Nausea or vomiting**							
No	33	1.0					
Yes	62	1.3 (0.4—4.2)	0.61				
**Unsteady or loss of balance**							
No	38	1.0					
Yes	57	0.9 (0.3—2.7)	0.91				
**Cephalic pressure**							
No	47	1.0					
Yes	48	1.1 (0.4—3.2)	0.83				
**Impaired speech**							
No	53	1.0					
Yes	42	0.3 (0.1—1.1)	**0.07**	0.6 (0.1—2.6)	0.49		
**Sensorimotor disturbance**							
No	59	1.0					
Yes	36	0.2 (0.0—0.8)	**0.03**	0.4 (0.1—2.4)	0.30		
**Loss of coordination**							
No	63	1.0					
Yes	32	0.8 (0.3—2.5)	0.68				
**Confusion**							
No	64	1.0					
Yes	31	0.8 (0.3—2.6)	0.76				
**Gait disturbance**							
No	72	1.0					
Yes	23	1.0 (0.3—3.3)	0.94				
**Reduced consciousness**							
No	83	1.0					
Yes	12	0.4 (0.0—3.2)	0.37				
**Tachycardia or POTS**							
No	60	1.0					
Yes	35	0.5 (0.1—1.6)	0.22				
**Sensitivity to smell**							
No	70	1.0					
Yes	25	0.1 (0.0—1.1)	**0.06**	0.2 (0.0—2.2)	0.20		

**Difficulty swallowing**							
No	75	1.0					
Yes	20	0.2 (0.0—1.6)	0.12				
**Runny nose**							
No	80	1.0					
Yes	15	0.0 (0.0— n/a)	1.00				
**Incontinence**							
No	85	1.0					
Yes	10	0.5 (0.1—4.1)	0.50				
**Sensitivity to taste**							
No	84	1.0					
Yes	11	0.4 (0.1—3.6)	0.43				
**Yawning**							
No	85	1.0					
Yes	10	0.0 (0.0—n/a)	1.00				
**Seizures**							
No	90	1.0					
Yes	5	0.0 (0.0—n/a)	1.00				
**Neck pain**							
No	12	1.0					
Yes	83	2.6 (0.3—21.9)	0.37				
**Interscapular pain**							
No	39	1.0					
Yes	56	1.0 (0.3—2.9)	0.99				
**Back pain**							
No	51	1.0					
Yes	44	1.0 (0.4—3.0)	0.95				
**Sensitivity to light**							
No	33	1.0					
Yes	62	1.0 (0.3—2.9)	0.96				
**Blurred vision**							
No	51	1.0					
Yes	44	1.4 (0.5—4.0)	0.55				

**Eye pain, fullness or pressure**							
No	57	1.0					
Yes	38	1.1 (0.4—3.1)	0.91				
**Double vision**							
No	71	1.0					
Yes	24	0.9 (0.3—3.1)	0.86				
**Watery eyes**							
No	85	1.0					
Yes	10	0.5 (0.1—4.1)	0.50				
**Tinnitus**							
No	26	1.0					
Yes	69	0.9 (0.3—2.8)	0.84				
**Sensitivity to sound**							
No	36	1.0					
Yes	59	0.8 (0.3—2.5)	0.76				
**Ear pain, fullness or pressure**							
No	42	1.0					
Yes	53	0.5 (0.2—1.4)	0.19				

OR = Odds ratio, CI = confidence interval, POTS = Postural orthostatic tachycardia syndrome

Bold values indicate statistical significance (p<0.1 for univariate analysis and p<0.05 for multivariate analysis). Model 2 and 3 of the stepwise regression models are not presented for brevity. Model 2 predictor variables included: time between seeing a healthcare professional and being diagnosed (p<0.001), sensorimotor disturbance (p=0.16) and sensitivity to smell (p=0.22). Model 3 included predictor variables included: time between seeing a healthcare professional and being diagnosed (p<0.001) and sensorimotor disturbance (p=0.08).

An odds ratio (OR) of more than 1 indicates that participants were more likely to rate their diagnosis as easy.

Table S2. Univariate analysis assessing predictors for rating obtaining treatment for a participant's "first" spinal CSF leak as easy

Covariate	N	Univariate logistic regression	
		**OR (95% CI)**	**P value**
**Sex**			
Male	16	1.0	
Female	74	1.7 (0.4—6.6)	0.43
**Age at diagnosis**			
≥40 years	49	1.0	
<40 years	41	1.3 (0.5—3.3)	0.61
**Time between seeing a healthcare professional and being diagnosed**			
≥3 months	55	1.0	
<3 months	35	1.2 (0.5—3.0)	0.75
**Time between diagnosis and obtaining treatment**			
≥1 month	37	1.0	
<1 month	52	5.2 (1.6—16.8)	**0.006**

OR = Odds ratio, CI = confidence interval

Bold values indicate statistical significance (p<0.1 for univariate analysis). Multivariate analysis was not required as only one predictor variable met statistical significance.

An odds ratio (OR) of more than 1 indicates that participants were more likely to rate obtaining treatment as easy.

Table S3. Univariate and multivariate backward stepwise logistic regression analysis assessing predictors for being currently symptomatic

Covariate	N	Univariate logistic regression		Multivariate logistic regression Model 1		Multivariate logistic regression Model 2	
		OR (95% CI)	P value	OR (95% CI)	P value	OR (95% CI)	P value
**Sex**							
Male	17	1.0					
Female	89	1.2 (0.4—3.8)	0.76				
**Age at diagnosis**							
≥40 years	60	1.0					
<40 years	46	0.5 (0.2—1.1)	**0.09**	0.5 (0.2—1.1)	0.09		
**Time between seeing a healthcare professional and being diagnosed**							
≥3 months	61	1.0					
<3 months	45	0.4 (0.1—0.9)	**0.03**	0.4 (0.1—0.9)	**0.03**	0.4 (0.1—0.9)	**0.03**
**Time between diagnosis and obtaining treatment**							
≥1 month	42	1.0					
<1 month	58	0.5 (0.2—1.2)	0.13				

OR = Odds ratio, CI = confidence interval

Bold values indicate statistical significance (p<0.1 for univariate analysis and p<0.05 for multivariate analysis).

An odds ratio (OR) of less than 1 indicates that a participant was less likely to be symptomatic at the time of survey completion.

Table S4. Univariate and multivariate backward stepwise linear regression analysis assessing predictors for EQ-VAS scores

Covariate	Univariate linear regression		Multivariate linear regression	
	**Unstandardised Coefficient B (95% CI)**	**P value**	**Unstandardised Coefficient B (95% CI)**	**P value**
Sex (Female)	3 (-12—18)	0.73		
Age at diagnosis (<40 years)	-15 (-25—-4)	**0.007**	-12 (-22—-2)	**0.02**
Time between seeing a healthcare professional and being diagnosed (<3 months)	8 (-3—20)	0.14		
Time between diagnosis and obtaining treatment (<1 month)	2 (-9—13)	0.69		
Headache present	-27 (-39—-15)	**<0.001**	-25 (-37—-13)	**<0.001**

OR = Odds ratio, CI = confidence interval

Bold values indicate statistical significance (p<0.1 for univariate analysis and p<0.05 for multivariate analysis)

A negative unstandardised coefficient B value indicates that for every relevant covariant, a lower EQ-VAS score was measured (i.e., lower self-perceived quality-of-life score)

Table S5. Univariate linear regression analysis assessing predictors for HIT-6 scores

Covariate	Univariate linear regression	
	**Unstandardised Coefficient B (95% CI)**	**P value**
Sex (Female)	-2 (-6—1)	0.20
Age at diagnosis (<40 years)	2 (-1—4)	0.21
Time between seeing a healthcare professional and being diagnosed (<3 months)	-2 (-4 —1)	0.27
Time between diagnosis and obtaining treatment (<1 month)	-1 (-4—1)	0.27

OR = Odds ratio, CI = confidence interval

No multivariate analysis was performed as no covariate reached significance in univariate linear regression (p<0.1)

A positive coefficient B value indicates that for every relevant covariant, a higher HIT-6 score was measured (i.e., the headache caused a greater impact)
